# Termination of DNA replication drives genomic instability via multiple mechanisms

**DOI:** 10.1093/nar/gkaf1519

**Published:** 2026-01-16

**Authors:** Daniel J Goodall, Juachi U Dimude, M Amin Hashemloo, Emma L Dunbar, Iren Grigoryan, Amy L Upton, Edward L Bolt, Christian J Rudolph

**Affiliations:** Division of Biosciences, College of Health, Medicine and Life Sciences, Brunel University of London, Uxbridge, UB8 3PH, United Kingdom; Division of Biosciences, College of Health, Medicine and Life Sciences, Brunel University of London, Uxbridge, UB8 3PH, United Kingdom; Division of Biosciences, College of Health, Medicine and Life Sciences, Brunel University of London, Uxbridge, UB8 3PH, United Kingdom; Department of Biochemistry, University of Wisconsin-Madison, Madison, WI 53706-1544, United States; Division of Biosciences, College of Health, Medicine and Life Sciences, Brunel University of London, Uxbridge, UB8 3PH, United Kingdom; Division of Biosciences, College of Health, Medicine and Life Sciences, Brunel University of London, Uxbridge, UB8 3PH, United Kingdom; School of Life Sciences, University of Nottingham, Medical School, Queen's Medical Centre, Nottingham, NG7 2UH, United Kingdom; Division of Biosciences, College of Health, Medicine and Life Sciences, Brunel University of London, Uxbridge, UB8 3PH, United Kingdom

## Abstract

Termination of DNA replication is a surprisingly complex process that contributes critically to genome stability and cell viability. And even though progress was made to establish the consequences that arise if termination is going awry, the precise molecular mechanisms of fork fusion events and the coordination with key factors that ensure that DNA replication is brought to a successful conclusion remain poorly understood. We therefore investigated replication termination in *Escherichia coli*, focusing specifically on the interplay between replication fork fusions and genomic stability, the Tus–*ter* replication fork trap, and key DNA-processing enzymes. By utilizing whole genome sequencing, immunoblotting, and recombination reporter assays, we demonstrate that local hyper-recombination is induced wherever forks meet and that the combined loss of factors such as RecG helicase and 3′ exonucleases causes extreme over-replication in the terminus region of the chromosome. Unexpectedly, cells lacking Tus exhibit elevated R-loop levels, revealing an unanticipated connection between the fork trap and R-loop metabolism. These findings underscore the complexity of replication termination and its central role in maintaining bacterial genome stability, while providing mechanistic insights with implications for understanding replication termination in more complex organisms and developing new antimicrobial strategies.

## Graphical Abstract

**Graphical Abstract:**
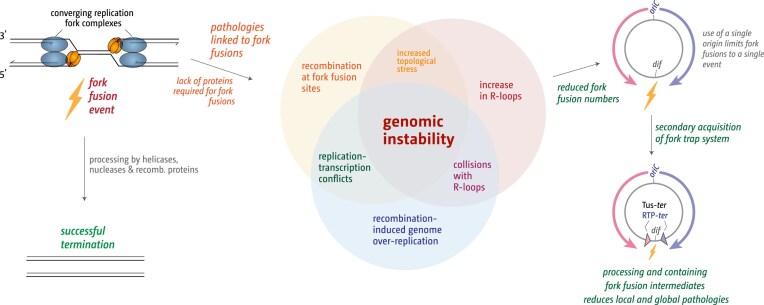

## Introduction

Faithful completion of chromosome replication is essential for genomic stability and cell viability. In bacteria, the normal mode of chromosomal DNA replication is initiated at a single origin, termed *oriC* [1], in stark contrast to the hundreds or even thousands of origins used in eukaryotic cells [2]. Once initiated, DNA synthesis proceeds bidirectionally until the two established replication forks fuse in the terminus region opposite *oriC* [3]. Termination, the final stage of DNA replication, is the critical conclusion step of the duplication process. Work from recent years has revealed that replication termination is a more intricate and tightly coordinated process than previously appreciated [4–7].

In some bacteria, including *Escherichia coli*, replication terminates within a defined chromosomal region known as the replication fork trap (RFT). This trap is defined by multiple *ter* sites which, when bound by the Tus terminator protein, form unidirectional barriers to replication fork progression. The Tus–*ter* complex functions through a ‘mousetrap’ mechanism, where the unwinding of DNA at the non-permissive face induces specific contacts that result in a ‘locked’ complex, efficiently blocking the oncoming replisome [8, 9]. In contrast, a replisome approaching from the permissive side simply displaces Tus, allowing the replisome to proceed. The RFT therefore permits forks to enter the terminus region but prevents them from leaving [4, 7, 10–14]. A recent phylogenetic analysis suggests that the fork trap system found in *E. coli* was domesticated from a plasmid-based precursor system [15], and its high degree of conservation across all *E. coli* lineages suggests that, once acquired, it confers a selective advantage [16].

RFT systems are found in only a limited number of bacterial species, indicating that many cells can replicate successfully without them. However, in *Bacillus subtilis*, a distinct fork trap system operates analogously to that of *E. coli* [4, 9, 17, 18], though neither the *ter* sequences nor the terminator protein RTP share sequence or structural similarity with the Tus–*ter* system. The *B. subtilis* terminator sites consist of two components—that A and B half-sites—with each half-site binding an RTP dimer [19]. Because RTP dimers bind more strongly to the B than to the A site, directional termination can be achieved: replication forks encountering the high-affinity B site are stopped, whereas those reaching the low-affinity A site can continue [20]. The lack of homology between the *E. coli* and *B. subtilis* systems suggests they arose through convergent evolution [9, 21], underscoring the functional importance of controlled fork termination.

What, then, makes fork trap systems important? Studies in *E. coli* have shown that fork fusion events can generate DNA intermediates that, if not properly processed, lead to pathological outcomes [22–32]. Proteins such as RecG helicase, 3′ exonucleases, DNA polymerase I, and others normally prevent such pathologies [22, 25–31, 33, 34]. In their absence, methods such as replication profiles established via whole-genome sequencing [35] show peaks of synthesis in locations where replication forks fuse, suggesting the local continuation of synthesis [23, 25–28]. The fork trap system efficiently restricts such over-replication to the terminus region, preventing its spread into the opposite replichore and allowing repair proteins to resolve aberrant intermediates [4].

A central factor in processing fork fusion intermediates appears to be RecG helicase [23, 25, 27, 36], which is conserved across nearly all bacterial species [37], as are many other proteins involved in termination. This widespread conservation may help to explain why many bacterial species do not possess a fork trap system: even without an RFT, these organisms retain the complete protein machinery for processing fork fusion intermediates. Any aberrant substrates can therefore be processed by RecG, 3′ exonucleases, and other proteins involved, allowing the successful completion of chromosome duplication. However, once acquired from a plasmid precursor, an RFT may provide an additional layer of protection by containing fusion-derived pathologies and promoting their efficient resolution, which results in a selective growth advantage [4, 15, 16].

If RFTs confer a selective advantage, the modest phenotypes associated with their loss are somewhat paradoxical. Although *∆tus* cells show a slight growth defect, the extension of their cell cycle is with <1 min minimal [16]. Similarly, chromosomal over-replication can be detected in *∆tus* single mutants, yet this phenotype remains similarly modest [23, 31]. These subtle effects seem at odds with the independent emergence and strict conservation of fork trap systems. The paradox is heightened by the fact that fork traps can also be detrimental: if one replisome stalls, the second cannot rescue it because it is blocked within the trap, a situation that can induce major chromosomal rearrangements and threaten cell viability [1, 4, 38, 39]. Thus, despite extensive studies, the physiological role and selective importance of RFTs have remained unclear.

Like many helicases, RecG acts on a broad range of DNA substrates, including Holliday junctions, fork-like structures, D-loops, and R-loops [40–49]. Consistent with RecG’s demonstrated capacity for R-loop resolution *in vitro*, genetic analyses reveal that cells simultaneously lacking RNase HI and RecG exhibit synthetic lethality [33, 50]. RNase HI, encoded by the *rnhA* gene, represents the principal enzyme governing R-loop homeostasis in *E. coli* through its degradation of RNA:DNA hybrid structures [51]. R-loops can interfere with replication, transcription, and DNA repair, and in eukaryotes they contribute to genome instability and cancer progression [52–54]. In bacteria, persisting R-loops can also act as primers for DNA replication, and in *E. coli* cells lacking RNase HI, this type of synthesis is strong enough to maintain chromosome duplication in the absence of origin firing [23, 55]. However, the pathological effects observed in the termination area in cells lacking RecG are unlikely to be triggered by R-loops [24], and a role of R-loops in termination of replication so far has not been described.

Here, we investigate how termination influences genomic stability and how specific proteins contribute to the processing of fork fusion intermediates, and we show that termination interfaces with R-loop metabolism. By using a recombination reporter cassette and fork fusion points that can be effectively switched ‘on’ and ‘off’, we demonstrate that elevated recombination frequencies arise locally within chromosomal regions where fork fusion events are activated in the *E. coli* chromosome. We demonstrate that the combined absence of proteins involved in termination, such as RecG helicase and 3′ exonucleases, results in a synergistic increase in the levels of over-replication observed in the termination area, highlighting that fork fusion events can trigger severe pathologies if termination intermediates are not adequately processed. Finally, we show that a link may exist between termination and R-loop metabolism, which also will impact genomic stability. Collectively, these findings demonstrate that fork fusion events compromise genomic stability through dual mechanisms: localized instability at fusion sites and elevated global R-loop accumulation. Functional RFT systems can partially mitigate these destabilizing effects. The fact that fork fusion events can impact genomic stability may help to explain the exclusive utilization of single replication origins in bacterial chromosomes. In addition, our findings raise fundamental questions regarding the mechanisms by which eukaryotic cells manage hundreds to thousands of termination events per cell cycle.

## Materials and methods

### Strains, media, and general methods

For *E. coli* K12 strains used in this study, see Supplementary Table S1. Strains were constructed via P1*vir* transductions [56] or by single-step gene disruptions [57]. Rich broth (LB) and agar were used in two different compositions. LB (Miller) contained 1% tryptone (Bacto™, BD Biosciences), 0.5% yeast extract (Bacto™, BD Biosciences), and 1% NaCl (Sigma–Aldrich) [58]. The pH was adjusted to 7.4. For plates, agar was added to a final concentration of 1%. For LB (Luria), 1% tryptone (Bacto™, BD Biosciences), 0.5% yeast extract (Bacto™, BD Biosciences), and 0.05% NaCl (Sigma–Aldrich) were used [59]. The pH was adjusted to 7.4, and for plates, agar was added to a final concentration of 1.5%. M9 minimal medium was purchased as a 5× concentrated stock (Sigma–Aldrich), which contained 15 g/l KH_2_PO_4_, 64 g/l Na_2_HPO_4_, 2.5 g/l NaCl, and 5.0 g/l NH_4_Cl. Before use, MgSO_4_, CaCl_2_, and glucose were added from sterile-filtered stock solutions to final concentrations of 2 mM, 0.1 mM, and 0.2%, respectively, according to the manufacturer’s recommendation.

### Recombination rate analysis

Mutation rates were measured using a Luria–Delbrück fluctuation test, as described previously [60], using a tandem repeat reporter cassette. Plasmid pRS316-*kankanMX4* [61] was used as template. The chloramphenicol resistance marker *cat*, flanked by *frt* sites from pKD3 [57], was polymerase chain reaction (PCR)-amplified, introducing restriction sites for BglII at both ends and cloned into the BglII site of pRS316-*kankanMX4*, generating plasmid pSLM001. Plasmid pSLM001 was used as template to amplify the *kankanMX4-<cat>* cassette, using primers that introduce 40 bp of homology to the integration locations of choice. For integration into the 4.53 and 3.39 Mbp locations an integration location between facing genes was chosen to avoid disruption of regulatory elements as much as possible. Thus, the cassette was integrated between *yjhR/yjhS* and *yhcS/tldD*. For integration near the natural fork fusion site at 1.54 Mbp, we selected a relatively large intergenic region between *yddJ* and *narU*, avoiding any interference with known regulatory elements in the region, as these two genes are not facing each other. For the rate and frequency measurements of these constructs, overnight cultures of the *E. coli* strains of interest were diluted 1:100 into 1 ml of LB (Miller) medium in 2 ml reaction tubes (Sarstedt) to achieve an initial *A*_600_ of 0.04. For each strain, 11 parallel cultures were grown at 37°C in a Thermomixer (Eppendorf) with shaking at 1000 rpm to an *A*_600_ of 0.4. One additional culture was grown alongside the fluctuation test cultures to allow determination of the cell density via *A*_600_ measurement. Viable titres were determined by spotting serial dilutions of the parallel *A*_600_ culture onto agar plates three times and the average colony count was used to represent the number of colonies for that dilution. Dilutions of 1 × 10^–5^ and 1 × 10^–6^ were used to avoid resolution issues for higher dilutions. When the target *A*_600_ was reached, cultures were centrifuged at 6000 × *g* for 5 min and resuspended in 100 µl LB (Miller) broth before plating onto LB (Miller) agar supplemented with 40 µg/ml kanamycin to select for mutants in which a reversion had taken place. Plates were incubated at 37°C for 24 h until colonies formed. Images were taken and colonies counted by using strict thresholding of images in ImageJ, followed by a particle count. Reversion frequencies were calculated by mutants/total cells, and mutation rates were calculated from colony counts using the Flan R package [62], implementing the Ma–Sandri–Sarkar maximum likelihood estimator generally accepted as the preferential method for determining rates from fluctuation data [63, 64]. By plating all cells of a grown culture, this method avoids dilution and pipetting errors as the total number of colonies counted represents the total number of mutants in the 1 ml culture grown. Unlike standard mutation rate analysis, where cultures are grown to saturation, this method uses cultures grown to a defined density, which is preferred, because the reversion rates using our system are orders of magnitude higher [36, 61] than that of standard mutation rates commonly used [65, 66].

### Marker frequency analysis by whole-genome sequencing

Marker frequency analysis by whole-genome sequencing was performed as described previously [35, 38, 67] with only minor modifications. Samples from cultures of a strain grown overnight in LB broth (Luria) were diluted 100-fold in fresh LB broth (Luria) and incubated with vigorous aeration until an *A*_600_ reached 0.4 at 37°C to ensure they were in exponential growth conditions. Cultures were then diluted a second time 100-fold in pre-warmed fresh broth and grown again until an *A*_600_ of 0.4 was reached. Samples from these exponential phase cultures were flash-frozen in liquid nitrogen at this point for subsequent DNA extraction. For wild type, incubation of the remaining culture was continued until several hours after the culture had saturated and showed no further increase in the *A*_600_. A further sample (stationary phase) was frozen at this point. DNA was then extracted using the GenElute Bacterial Genomic DNA Kit (Sigma–Aldrich). Marker frequency analysis was performed using Illumina HiSeq 2500 sequencing (fast run) to measure sequence copy number. FastQC was used for a basic metric of quality control in the raw data. Bowtie2 was used to align the sequence reads to the reference. Samtools was used to calculate the enrichment of uniquely mapped sequence tags in 1 kb windows.

For presentation of the data as a marker frequency replication profile, the raw read counts for each construct were divided by the average of all read counts across the entire genome to correct for the somewhat different absolute numbers of aligned reads in the various samples. The normalized read count values for each exponentially growing sample were then divided by the corresponding normalized read count value from a stationary (non-replicating) sample. This division ‘cleans’ the raw data significantly, because data points that are outliers caused by technical aspects (precise sequence environment interfering with library preparation or similar issues) will be similarly distorted both in the exponential and the stationary samples.

### Marker frequency analysis in cells with synthetically lethal genotypes

Marker frequency analysis in cells with synthetically lethal genotypes, such as *∆recG ∆rnhA*, was done in the presence of a covering plasmid carrying a functional copy of *recG* under the p*araBAD* promoter. Overnight cultures of the strains of interest were grown, either in LB broth or M9 minimal medium, with 0.2% arabinose to keep expression of the functional copy of *recG* switched on. For the experiment the overnight culture was diluted 1:100 into 100 ml of either LB broth or M9 minimal medium supplemented with 0.2% arabinose. Cultures were grown in 250 ml polycarbonate Erlenmeyer flasks (Corning) until they reached an *A*_600_ of 0.2. Cultures were washed twice and resuspended in 100 ml of either LB broth or M9, either containing 0.2% arabinose to induce *recG* expression or 0.2% glucose to switch *recG* expression off by catabolite repression [68]. Ten millilitre samples were taken at the times indicated and flash-frozen in liquid nitrogen. Genomic DNA was then extracted for whole-genome sequencing as described earlier.

### R-loop detection by dot blot

R-loop formation was analysed by dot blotting, as described [69, 70]. Samples from cultures of a strain grown overnight in LB broth (Miller) were diluted 100-fold in fresh LB broth (Miller) and incubated with vigorous aeration until an *A*_600_ reached 0.4 at 37°C to ensure they were in exponential growth conditions. Cultures were then harvested at 5000 × *g* for 5 min and resuspended in ice-cold PBS (Sigma–Aldrich). Genomic DNA was extracted using the Monarch Genomic DNA Purification Kit (NEB) and quantified using a BioDrop spectrophotometer (Harvard Biosciences). Samples were diluted to 20 ng/µl in elution buffer. For dot blotting, 5 µl of DNA was spotted onto Hybond N^+^ nitrocellulose membrane (GE Healthcare). DNA was UV crosslinked to the membrane using a UV stratalinker 1800 (Stratagene) at 120 mJ/cm². Membranes were blocked for 1 h at room temperature with 2% non-fat milk in PBS. Blocked membranes were incubated for 2 h at room temperature with the S9.6 antibody (Kerafast) diluted 1:5000 in 0.1% PBS 0.05% Tween^®^ 20, followed by five washes for 5 min with 0.1% PBS 0.05% Tween^®^ 20. Membranes were then incubated for 1 h at room temperature with HRP-conjugated goat anti-mouse secondary antibody diluted 1:5000 in 0.1% PBS Tween^®^ 20, followed by a further six washes for 5 min. Chemiluminescence detection was performed by incubating membranes for 5 min with ECL reagent (Bio-Rad) at room temperature and imaging on a G:Box Chemi XX6 (Syngene) with a 30 s exposure. Dot intensities were quantified using ImageJ software. Background subtraction was performed by measuring intensity of a blank region of membrane and subtracting this from each dot intensity value. R-loop levels were normalized to the level in wild-type cells. The enzymes RNase A, RNase III, and RNase HI were all purchased from NEB, and gDNA samples were incubated for 60 min at 37°C with these enzymes before being transferred to the nitrocellulose membrane.

### R-loop detection by dot blot in synthetically lethal strains

For the analysis of the synthetically lethal *∆recG ∆rnhA* genotype, we performed experiments in the presence of a covering plasmid carrying a functional copy of *recG* under the p*araBAD* promoter. In this case overnight cultures were grown in LB (Miller) with 0.2% arabinose to keep expression of the functional copy of *recG* switched on. For the experiment the overnight culture was diluted 1:100 into LB (Miller) and grown to an *A*_600_ of 0.2. The cultures were washed twice with M9 minimal medium and resuspended in 100 ml LB (Miller) either containing 0.2% arabinose (expression of the *recG* gene is maintained) or in LB (Miller) containing 0.2% glucose (expression of the *recG* gene is repressed by catabolite repression) [68]. Two millilitre samples were taken at the times indicated and flash-frozen in liquid nitrogen. Genomic DNA extraction and detection of R-loop levels via dot blot were then performed as described earlier.

### Fluorescence microscopy of synthetically lethal strains

For the analysis of replication complexes in the synthetically lethal *∆recG ∆rnhA* genotype, we performed experiments as before in the presence of a covering plasmid carrying a functional copy of *recG* under the p*araBAD* promoter. In this case overnight cultures of strains also carrying a *YPet-dnaN* allele (see Supplementary Table S1) were grown in LB (Miller) with 0.2% arabinose to keep expression of the functional copy of *recG* switched on. For the experiment the overnight culture was diluted 1:100 into LB (Miller) and grown to an *A*_600_ of 0.2. 2 × 2 ml aliquots were washed twice with LB (Miller) and resuspended in 2 ml fresh LB (Miller). To one culture glucose was added to a final concentration of 0.2% (expression of the *recG* gene is repressed by catabolite repression) [68], while arabinose to a final concentration of 0.2% was added to the other. One microlitre samples were taken at the times indicated and pipetted onto an agarose pad, which was then air-dried. For generation of pads, a 65 µl (15 × 16 mm) GeneFrame (Thermo Scientific) was added to a conventional microscopy slide. One per cent of SeaKem LE agarose (Lonza) was added to 1× M9 minimal medium (diluted from a 5× stock, Sigma–Aldrich) and heated until the agarose was completely dissolved. Ninety-five microlitres of the solution was added into the GeneFrame chamber, and the chamber sealed immediately with a conventional microscopy slide. Once set, the top slide was removed and the agarose pad air-dried for 15 min at room temperature and used immediately. Once the sample was added and air-dried, the GeneFrame chamber was sealed by adding a 22 × 22 mm cover slip. Visualization was done using a Ti-U inverted microscope (Nikon) with a CFI Plan Fluor DLL 100× objective (Nikon) and an ORCA Flash 4.0 LT plus camera (Hamamatsu). Phase contrast images were taken using a pE-100 single LED wavelength source (CoolLED). For fluorescence, the pE-4000 illumination system (CoolLED) was used. The relevant filter for visualization of YPet was Zeiss filter set 46. Images were captured using the NIS Elements-BR software V4.52 (Nikon). Standard imaging parameters were 16-bit without binning, with standard exposure times of 100 ms for phase contrast and 1.5 s for YPet-DnaN images.

### Cell length analysis

For the analysis of cell lengths in brightfield images, the open-source program Fiji/ImageJ (https://fiji.sc/) and the free plugin MicrobeJ (https://www.microbej.com/) were used [71]. MicrobeJ automatic detection was performed for cell segmentation and cell length analysis. Following the automatic detection, some manual adjustments were used to verify cell outline detection and, if necessary, adjustments were made to restrict the analysis to individual cells. Cell length data were then copied to SigmaPlot (V16, Grafiti LLC). Data representation was achieved by using the violin plot function on the raw cell length data within SigmaPlot.

### Statistical analysis

Recombination frequencies were calculated by dividing the number of mutants by the total number of viable cells. Mutation rates were calculated from colony counts (see Supplementary Fig. S1) using the Flan R package [62], implementing the Ma–Sandri–Sarkar maximum likelihood estimator, a preferential method for determining rates from fluctuation data [63, 64]. By plating whole cultures this method avoids dilution and pipetting errors as the total number of colonies counted represents the total number of mutants in the 1 ml culture used for the experiment. Unlike standard mutation rate analysis, where cultures are grown to saturation, this method uses cultures grown to a defined optical density, which is particularly useful as the reversion rates using the tandem repeat reporter system are orders of magnitude higher than traditional spontaneous point mutations. For representation of rates, bar graphs show the average of 4 independent rate experiments, with error bars showing standard error of the mean (SEM). An ANOVA test was performed using the Analysis of Variance function (aov()) on each group of data points to determine *P-*values. For R-loop quantification, dot intensities were quantified using ImageJ software. Background subtraction was performed by measuring intensity of a blank region of membrane and subtracting this from each dot intensity value. R-loop levels were normalized to the level in wild–type cells. For determination of *P-*values a one-way ANOVA test was performed (aov()). The data were plotted in R using tidyverse and ggplot2. For generation of the LOESS regression from the replication profiles of synchronized *dnaA46* cells in the presence and absence of Tus, the ‘smoothing 2D data’ functionality within SigmaPlot (V16, Grafiti LLC) was employed, using LOESS as smoothing method.

## Results

### Fork fusion events induce local hyper-recombination

**Figure 1.**
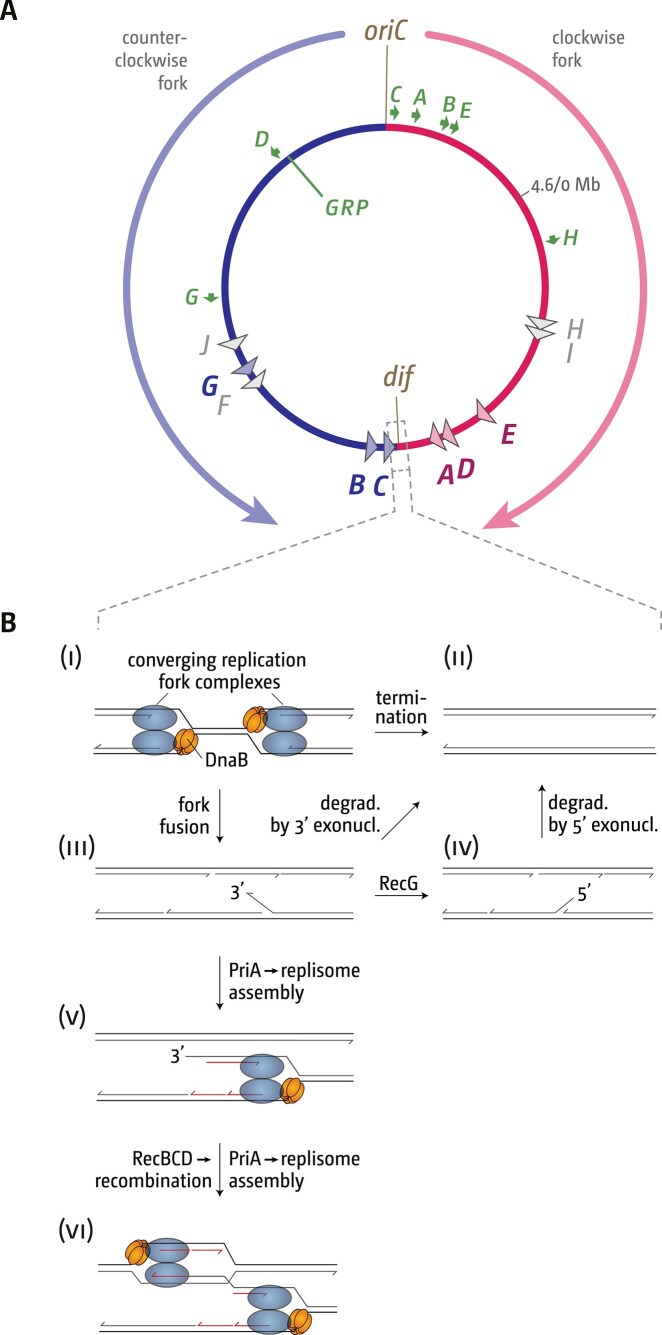
Chromosome structure of the *Escherichia coli* chromosome and schematic detailing the pathologies triggered by fork fusion events in the termination area. (**A**) Schematic representation of the *E. coli* chromosome. Two replication forks are initiated at a single origin termed *oriC* and move in opposite directions along the DNA until they approach one another and fuse within the terminus region opposite *oriC*. An RFT is formed in the terminus region via terminator sequences (*terA–J*), which are arranged as two opposed groups, with the pink terminators orientated to block movement of the clockwise replication fork and the blue terminators orientated to block the counter-clockwise fork. For *ter* sites *F, H, I*, and *J*, no binding of the Tus terminator protein was found [10], and for this reason these are shown in grey. The location of the *dif* chromosome dimer resolution site is marked. Locations of the *rrn* operons, which are particularly highly transcribed under fast growth conditions, are shown by green arrows, with the arrow pointing in the direction in which transcribing RNA polymerase molecules travel. ‘GRP’ indicates the location of a cluster of genes encoding ribosomal proteins, almost all of which are transcribed co-directionally with replication. (**B**) Schematic illustrating how replication fork fusions might trigger over-replication in the termination area and how this is normally prevented by proteins such as RecG and/or 3′ exonucleases. Note that the formation of a 3′ flap can occur at both forks. For simplicity the schematic shows only one such reaction. See text for further details.

Based on genetics and cell biology data [22, 23, 25–28, 33], we proposed a hypothesis of how termination in bacteria can result in a number of pathologies (Fig. 1). We proposed that the fusion of two forks can result in the formation of 3′ flap intermediates, as the helicase of one fork interacts with the opposing leading-strand polymerase. Such 3′ flaps are normally processed by RecG helicase, which converts them into 5′ flaps, or degraded by one of the several 3′ exonucleases present in *E. coli* (Fig. 1B iii and iv). In addition, 3′ flap structures are also a substrate for the replication restart protein PriA, which may reload a functional replisome [72]. Progression of such replisomes will over-replicate an already fully replicated chromosome (Fig. 1B v). In addition, replication will result in the formation of a dsDNA end, which may engage in homologous recombination (HR), which can trigger recombination-dependent replication, thereby exacerbating the over-replication (Fig. 1B vi). This model explains the over-replication observed in the termination area if proteins such as RecG or 3′ exonucleases are missing [4, 22, 23, 26–28, 33].

**Figure 2.**
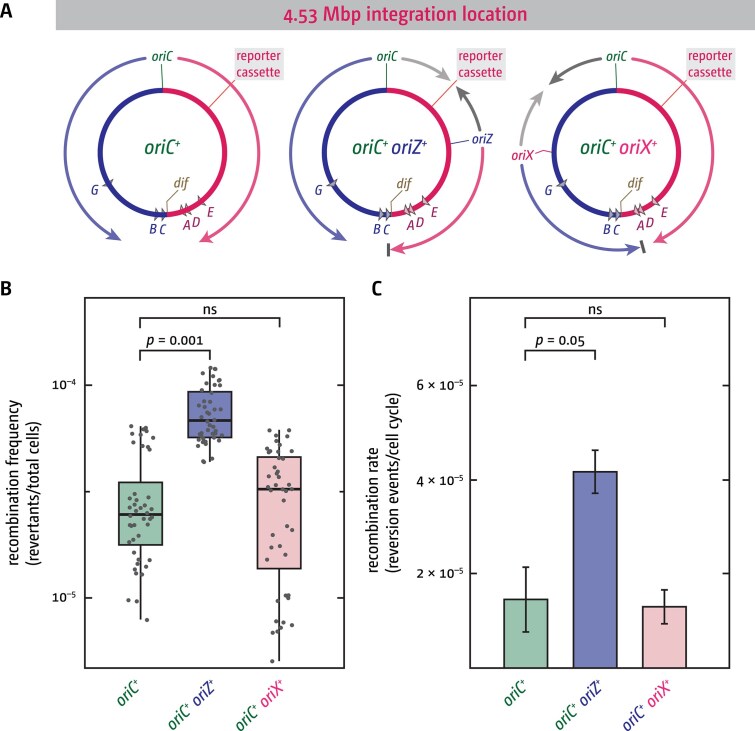
Fork fusion events in an ectopic location trigger increased recombination. (**A**) Overview of the *E. coli* chromosome, highlighting the integration site of the tandem repeat reporter cassette at 4.53 Mbp, integration sites of the additional ectopic replication origins *oriZ* and *oriX*, as well as the resulting replichore configuration and termination sites. Due to the shorter replichore lengths resulting from ectopic origin integration, the fork initiating from the ectopic origin and travelling in the normal direction always reaches the termination area earlier than the fork coming from *oriC* and is arrested at Tus–*ter* complexes [38], [39], indicated by the grey bar. (**B**) Tandem repeat reversion frequencies measured in cells with only the native *oriC* (*oriC^+^*) as well as cells that carry either the additional ectopic *oriZ* (*oriC^+^ oriZ^+^*) or *oriX* (*oriC^+^ oriX^+^*), effectively switching fork fusion events in the location where the tandem repeat reporter cassette is integrated on and off. Data from 44 individual cultures are shown for each strain, generated across 4 independent experiments. An ANOVA test was performed on each group of data points to determine *P-*values, which are indicated above the data points. (**C**) Tandem repeat reversion rates were calculated at the end of each experiment from the median data points of individual experiments shown in panel (B). Bar graphs show an average of the 4 rates, with the error bars showing the standard error of the mean (SEM). An ANOVA test was performed on the data to determine *P*-values, which are indicated above the data points. The strains used were DG010 (*oriC^+^*), DG024 (*oriC^+^ oriX^+^*) and DG026 (*oriC^+^ oriZ^+^*). All had the tandem repeat reporter cassette integrated at *yjhR* near position 4.53 Mbp of the chromosome.

Our working model predicts that any region where forks fuse will show increased recombination frequencies (Fig. 1B vi). Indeed, the involvement of key recombination proteins in termination was shown by us and others [23, 25, 29, 30, 34, 73, 74]. However, while these studies imply that it might be the fork fusion process that may trigger homologous recombination, they do not demonstrate this point directly. To measure whether fork fusions can trigger increased recombination frequencies, we used *E. coli* strains in which an additional ectopic origin termed *oriZ* is integrated into the chromosome. In these cells an additional termination event occurs between the native and the ectopic origin at ~4.53 Mbp [38, 39]. Thus, in wild-type cells termination is ‘switched off’ in this ectopic location, whereas in the presence of *oriZ*, fork fusions are ‘switched on’ (Fig. 2A). To measure recombination events, we integrated a previously used reporter cassette that contains a short 266 bp tandem repeat [36, 61] at the 4.53 Mbp location of the chromosome. The direct tandem repeat is located within a kanamycin resistance marker, thereby interrupting its open reading frame (Supplementary Fig. S1A). Any event that results in the clean deletion of one of the two repeats will restore resistance (Supplementary Fig. S1B), which can be easily scored on medium containing kanamycin [36, 61] (Supplementary Fig. S1C).

Recombination frequencies in wild-type cells (*oriC^+^*) with this chromosomally integrated construct were around 5 × 10^–5^ mutants/total number of cells, with a reversion rate of ~1.5 × 10^–5^ events per cell cycle (Fig. 2B). As predicted, integration of *oriZ* (*oriC^+^ oriZ^+^*) resulted in increased recombination, showing a frequency of ~8 × 10^–5^ mutants/total number of cells (*P* = .005), and a recombination rate of ∼4 × 10^–5^ events per cell cycle (*P* = .05) (Fig. 2C and Supplementary Fig. S1B). The recombination rate was increased 2.6-fold compared to the single-origin counterpart. In cells lacking RecA recombinase a significant decrease of the recombination frequencies was observed (Supplementary Fig. S2A and B), but they were not entirely eliminated, as observed before [36, 75–77]. In contrast, deletion of *recG* increases recombination frequencies ∼1.5-fold (Supplementary Fig. S2C), in line with the over-replication observed at this fork fusion site in *oriC^+^ oriZ^+^* cells lacking RecG [25].

To exclude the possibility that the observed increase is triggered purely by the presence of the additional ectopically located origin, we constructed a strain with the tandem repeat reporter cassette integrated in the same ectopic location (4.53 Mbp), but with an ectopic origin termed *oriX* integrated in the opposite replichore (*oriC^+^ oriX^+^*). Thus, while these cells also contain an additional ectopic origin, fork fusions occur between *oriX* and the native *oriC* (Fig. 2A) in a different region of the chromosome from where our reporter cassette is located—in the location of the reporter cassette fork fusions are ‘switched off’. In these strains we did not detect major changes of recombination events: recombination frequencies and rates were not increased (Fig. 2B and C).

**Figure 3.**
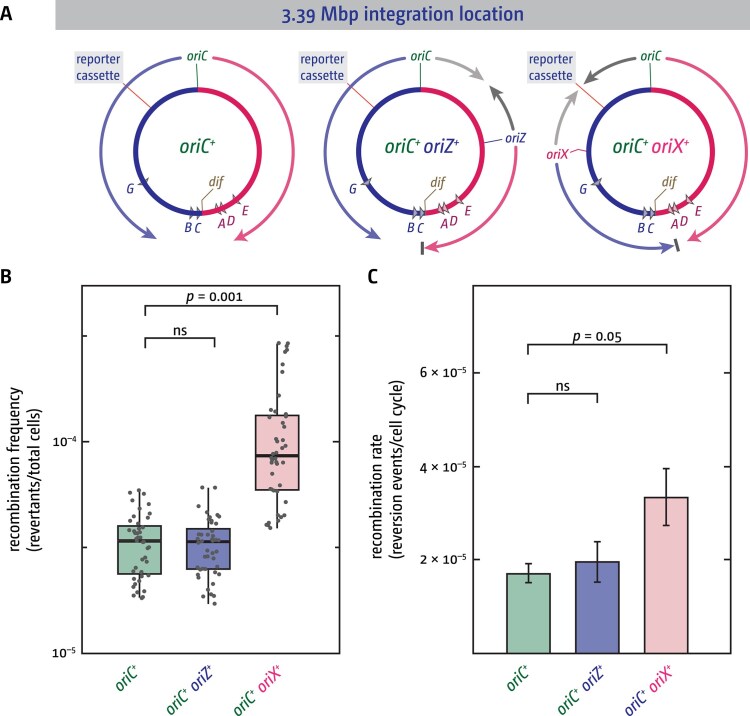
Fork fusion events trigger increased recombination regardless of the position of the fork fusion point. (**A**) Overview of the *E. coli* chromosome, highlighting the integration site of the tandem repeat reporter cassette at 3.39 Mbp, integration sites of the additional ectopic replication origins *oriZ* and *oriX*, as well as the resulting replichore configuration and termination sites. Due to the shorter replichore lengths resulting from ectopic origin integration, the fork initiating from the ectopic origin and travelling in the normal direction always reaches the termination area earlier than the fork coming from *oriC* and is arrested at Tus–*ter* complexes [38, 39], indicated by the grey bar. (**B**) Tandem repeat reversion frequencies measured in cells with only the native *oriC* (*oriC^+^*) as well as cells that carry either the additional ectopic *oriZ* (*oriC^+^ oriZ^+^*) or *oriX* (*oriC^+^ oriX^+^*), effectively switching fork fusion events in the location where the tandem repeat reporter cassette is integrated on and off. Data from 44 individual cultures are shown for each strain, generated across 4 independent experiments. An ANOVA test was performed on each group of data points to determine *P-*values, which are indicated earlier in the data points. (**C**) Tandem repeat reversion rates were calculated at the end of each experiment from the median data points of individual experiments shown in panel (B). Bar graphs show an average of the 4 rates, with the error bars showing the standard error of the mean (SEM). An ANOVA test was performed on the data to determine *P-*values, which are indicated above the data points. The strains used were DG059 (*oriC^+^*), DG061 (*oriC^+^ oriX^+^*), and DG062 (*oriC^+^ oriZ^+^*). All had the tandem repeat reporter cassette integrated at *tldD* near position 3.39 Mbp of the chromosome.

To rule out that the specific integration location of the reporter cassette somehow influenced recombination events, we integrated the cassette into the fork fusion location between *oriC* and *oriX* (Fig. 3A). If fork fusions are responsible for triggering an increase in the number of recombination events, then we would expect a mirror to the situation we observed with the first integration location: recombination should be increased in *oriC^+^ oriX^+^* strains but remain unchanged in *oriC^+^ oriZ^+^* strains.

This is precisely what we observed. The recombination frequencies are significantly increased in *oriC^+^ oriX^+^* cells in comparison to wild-type (*oriC^+^*) cells (*P* = .001), and the tandem repeat reversion rate was increased by a factor of ∼2, whereas both reversion frequencies and rates were not changed in *oriC^+^ oriZ^+^* cells (Fig. 3B and C). In combination, these data rule out that any of the increases observed are caused purely by the integration of an ectopic origin or that a specific location might be responsible for higher tandem repeat reversion rates. Thus, the data shown suggest that the head-to-head fusion of two replication fork complexes can directly trigger increased local recombination events. This effect is detectable in wild-type cells with fully functioning DNA repair systems, showing that increased local recombination rates are not triggered by the absence of factors such as RecG or 3′ exonucleases. Nonetheless, loss of RecG enhances this phenotype, indicating that RecG normally helps to limit recombination at these sites (Supplementary Fig. S2C).

Increased recombination could, in theory, result from double-stranded DNA breaks formed when additional rounds of synthesis catch up with pre-existing forks. Such head-to-tail collisions occur more frequently in cells that over-initiate at *oriC* and can generate catastrophic breaks [78]. However, this mechanism appears unlikely in our experimental system. The doubling time of cells carrying two origins differs only slightly from wild type (20.6 versus 19.9 min) [38], indicating normal growth without evidence for the accumulation of toxic intermediates. Moreover, replication profiles show no signs of increased origin firing in double-origin strains compared to wild type [38, 39], arguing against repeated initiation events that would be required for such run-off products to form. In addition, we analysed the level of active synthesis in cells by using a fluorescently labelled version of the sliding clamp, DnaN-YPet, to investigate whether the number of fluorescent foci is significantly increased in *oriC^+^ oriZ^+^* cells. In most wild-type cells between 2 and 4 DnaN-YPet foci are visible when using conventional wide-field microscopy. In double-origin cells, the foci distribution exhibits a modest broadening, with a subset of cells displaying 4 and 5 foci (Supplementary Fig. S3A and B). Nevertheless, the median remains unchanged at 3 foci per cell, while the mean increases from 2.8 in wild type to 3.2 in *oriC^+^ oriZ^+^* cells. Thus, even though twice the number of replisomes are initially established in cells with two replication origins [39, 79], replichore lengths between *oriC* and *oriZ* are quite short (∼0.6 Mbp), and the mild shift in foci numbers is in line with the idea that replisomes do not persist over long periods of time. Together with the evidence from replication profiling [38], there is no indication that origins in these strains are over-initiating. Run-off replication will therefore not contribute significantly to the observed increase in recombination events.

We previously proposed that the Tus–*ter* RFT found in some bacterial species such as the Enterobacteriaceae provides a system that can protect the cells from the pathologies triggered by fork fusions [4, 16]. Therefore, we wanted to investigate whether recombination is influenced by the RFT. If the fork trap confers a protective effect, then Tus terminator protein deficiency should result in elevated tandem repeat reversion events, reflecting increased genomic instability.

**Figure 4.**
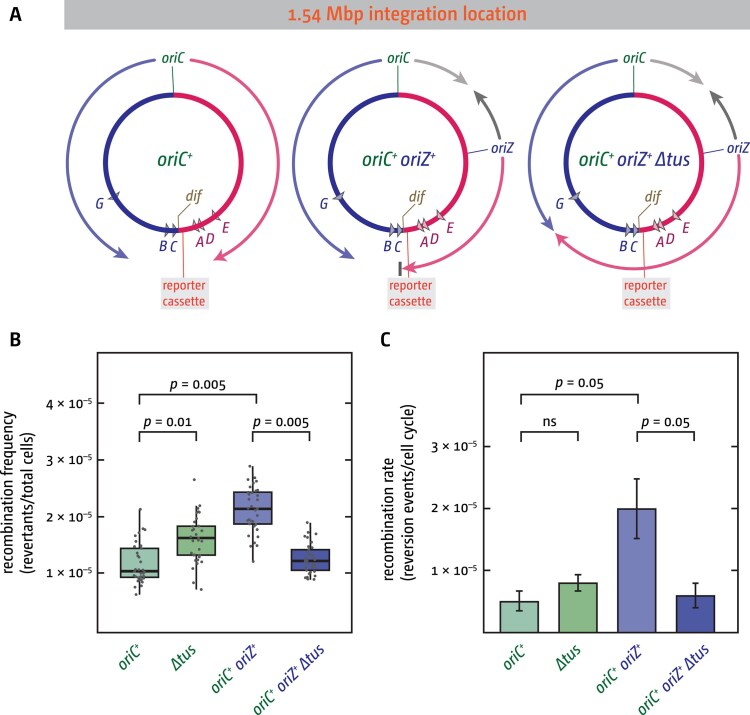
Effect of the presence or absence of the RFT on recombination events in the native termination area. (**A**) Overview of the *E. coli* chromosome, highlighting the integration site of the tandem repeat reporter cassette within the native termination area at 1.54 Mbp. The locations of the *ter* sites bound by the Tus terminator protein in normally growing cells [10] are shown. In the absence of Tus replication fork complexes will proceed through *ter* sites in any orientation unhindered. The integration location of *oriZ* is highlighted, as well as the resulting replichore configuration and termination sites. (**B**) Tandem repeat reversion frequencies measured in cells in the presence or absence of Tus terminator protein, as well as the presence and absence of the additional ectopic *oriZ*. Data from 33 individual cultures are shown for each strain, generated across 3 independent experiments. An ANOVA test was performed on each group of data points to determine *P-*values, which are indicated above the data points. (**C**) Tandem repeat reversion rates were calculated at the end of each experiment from the median data points of individual experiments shown in panel (B). Bar graphs show an average of the 3 rates, with the error bars showing the standard error of the mean (SEM). An ANOVA test was performed on the data to determine *P-*values, which are indicated above the data points. The strains used were SLM1042 (*oriC^+^*), DG011 (*∆tus*), DG028 (*oriC^+^ oriZ^+^*), and DG033 (*oriC^+^ oriZ^+^ ∆tus*). All had the tandem repeat reporter cassette integrated near *narU* at position 1.54 Mbp of the chromosome.

We integrated the tandem repeat reporter cassette near the native fork fusion point within the chromosomal termination area (Fig. 4A) and determined tandem repeat deletion frequencies and rates in the presence and absence of Tus. Consistent with our hypothesis, Tus deficiency significantly increased reversion frequencies (*P* = .01) (Fig. 4B). Although reversion rates exhibited a 1.5-fold elevation, this change was not statistically significant (Fig. 4C). This discrepancy reflects the distinct analytical approaches: frequency calculations utilize all experimental data, whereas rate determinations employ only the median value from the 11 cultures grown in parallel [65].

We also measured reversion rates and frequencies in *oriC^+^ oriZ^+^* strains. In these cells, the fork from *oriZ* encounters the RFT earlier than the *oriC*-derived fork, leading to frequent arrest at Tus–*ter* complexes [1, 38, 39]. Because *ter* sites were shown to act as recombination hotspots [34, 80], likely reflecting the recombinational processing of arrested replication forks, we expected increased recombination in the termination area because of the high frequency of forks stalling at Tus–*ter* complexes. This increase should be Tus-dependent, as removing Tus would remove the obstacle to DNA synthesis.

Consistent with this hypothesis, *oriC^+^ oriZ^+^* cells exhibited elevated recombination rates in the termination region in a Tus-dependent manner (Fig. 4B and C). Notably, recombination frequencies in *oriC^+^ oriZ^+^ ∆tus* cells were marginally reduced compared to *∆tus* single mutants, although this difference did not achieve statistical significance. However, we believe it might be an important indicator. We showed before that in *oriC^+^ oriZ^+^* cells lacking Tus, termination is shifted significantly into the left-hand replichore, away from the native termination area [38]. This means that in *oriC^+^ oriZ^+^ ∆tus* cells forks do not fuse near the reporter cassette located within the termination area any longer. Thus, if fork fusion events trigger increased recombination frequencies, then this shift away from where our reporter cassette is located should result in a decrease of recombination events, which is indeed what we observed (Fig. 4B and C). Although this non-significant difference limits firm conclusions, we propose that spatial displacement of fork fusion sites relative to the reporter cassette accounts for the diminished recombination frequency observed in *oriC^+^ oriZ^+^ ∆tus* cells (Fig. 4B and C).

The data presented are in line with the idea that fork fusion events in the termination area trigger increased recombination frequencies. A role of the RFT in limiting these negative consequences is less clear, but the trends seen are compatible with this interpretation. However, even if the assumption of a protective effect by the RFT is correct, having such a system in place comes at a price: Tus–*ter* complexes are a strong obstacle to replication, and these complexes become a hotspot for recombination themselves if forks are held for any length of time [34, 80]. However, while forks arrested at Tus–*ter* complexes are easily detected in normally growing wild-type cells, this still remains a rare event [81], in line with the observation that the majority of forks fuse in the same location regardless of the presence or absence of Tus [11, 23]. Thus, the fork trap mechanism does not define where termination takes place but is likely located near fork fusion points to limit and contain pathologies associated with fork fusions.

### Cells lacking RecG helicase and 3′ exonucleases show extreme over-replication of the chromosomal termination region

Our genetics and molecular cell biology data indicate that a 3′ flap structure (Fig. 1B iii) is a central intermediate that can arise as replication forks fuse [23, 24, 26, 33]. *Escherichia coli* contains a variety of exonucleases with 3′–5′ activity [82]. We previously investigated the roles of ExoI (encoded by *xonA*), ExoVII (*xseA*), and SbcCD (*sbcC* and *sbcD*). We and others showed that inactivation of single genes resulted in only minor termination defects, while the combined deletion of several 3′ exonucleases resulted in increasing levels of over-replication in the termination area [23, 26, 28]. We also showed that inactivating all three major 3′ exonucleases, ExoI, ExoVII, and SbcCD, was synthetically lethal in combination with the inactivation of RecG helicase [33]. Our interpretation was that the increasing inability to process 3′ flap structures, either via degradation (3′ exonucleases) or helicase activity (RecG), results in the accumulation of intermediates, which are toxic and eventually lead to cell death.

**Figure 5.**
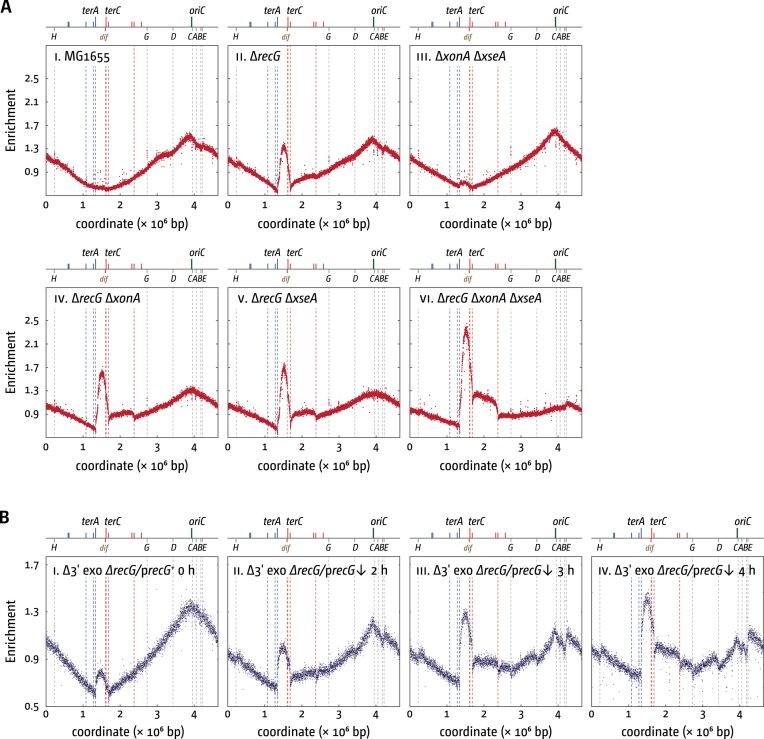
Over-replication in the termination area of *E. coli* cells lacking RecG helicase and 3′ exonuclease proteins. (**A**) The number of sequence reads (normalized against reads for a stationary-phase wild-type control) is plotted against the chromosomal location. A schematic representation of the *E. coli* chromosome showing positions of *oriC* and *ter* sites (above) as well as *dif* and *rrn* operons *A–E, G*, and *H* (below) is shown above the plotted data. Sequencing templates were isolated from MG1655 (wild type), AU1015 (*∆recG*), SLM1203 (*∆xonA ∆xseA*), RCe667 (*∆xonA ∆recG*), JD1587 (*∆xseA ∆recG*), and JD1579 (*∆xonA ∆xseA ∆recG*). (**B**) Cells carrying deletions of the genes for RecG helicase and the 3′ exonucleases ExoI, ExoVII, and SbcCD (*∆xonA, ∆xseA*, and *∆sbcCD*, respectively), with the deletion of *recG* covered by a plasmid with an arabinose-inducible *recG* gene, were grown in M9 minimal medium with arabinose to maintain *recG* expression. Cells were then washed and resuspended in M9 minimal medium with glucose instead of arabinose, switching the expression of the *recG* gene off (downwards pointing arrow). Samples were taken at the intervals shown, and replication profiles were generated as described in panel (A). The strain used was JD1521 (*∆xonA ∆xseA ∆sbcCD ∆recG* p*recG^+^*).

The pathological phenotypes of cells deficient in either RecG or 3′ exonucleases have been well characterized. Our working model predicts a synergistic interaction if both RecG and single 3′ exonucleases are missing. However, double mutants have so far remained unexamined. Replication profiles established via whole genome sequencing showed that cells lacking RecG have moderate levels of over-replication in the terminus area, as observed before [23–25] (Fig. 5A ii). Cells lacking single 3′ exonuclease genes show effects that are barely detectable (Supplementary Fig. S4), with only the combined absence of at least two 3′ exonucleases resulting in small amounts of over-replication (Fig. 5A iii), as observed [26].

Remarkably, inactivation of even a single exonuclease gene in *∆recG* cells produces over-replication peaks within the termination area that surpass those observed at *oriC* (Fig. 5A v and Supplementary Fig. S4), in line with the prediction of a synergistic interaction. This effect becomes further amplified when two 3′ exonucleases are deleted, generating even more extensive levels of over-replication in the termination area (Fig. 5A vi). These dramatic effects represent major pathologies that arise as part of replication termination. However, it is noteworthy that despite these pathologies, we did not notice drastic growth defects when constructing and growing the constructs.

Given the strong effects, we wanted to establish whether other additional effects contribute to the lethality observed in cells lacking RecG and the three major exonucleases ExoI, ExoVII, and SbcCD. To do so, we generated a strain lacking RecG and all three exonucleases, with the chromosomal deletion of the *recG* gene covered by a plasmid with a wild-type copy of the *recG* gene under the control of the p*araBAD* promoter. Due to inconsistent growth of this strain in rich medium, despite *recG* expression, we employed M9 minimal medium, which supported robust growth under these conditions. This is in line with previous results showing increased viability of cells lacking all three exonucleases and RecG on minimal medium [33]. Cells were then grown in medium with arabinose to early exponential phase, washed, and resuspended in medium with either arabinose or glucose, the latter repressing transcription of the plasmid-encoded *recG* gene (see the ‘Materials and methods’ section). Samples were taken as indicated, and replication profiles were generated via whole genome sequencing (Fig. 5B).

In line with our results shown in Fig. 5A, the strongest effect observed was a substantial peak of over-replication in the termination area (Fig. 5B), similar to the effects observed in double and triple mutants (Fig. 5A). We did, however, notice other distortions of the replication profiles as well, in particular at the highly transcribed *rrn* operons, which showed significant reductions in the marker frequencies at later time points. Thus, while the over-replication triggered by the absence of both RecG and 3′ exonucleases is extensive and can have lethal consequences [25], other effects may well contribute towards the lethality, in line with the idea that both RecG helicase and 3′ exonucleases have been proposed to be involved in a variety of cellular processes [82–86].

### The combined deletion of RecG and RNase HI does not result in major observable termination defects

Cells lacking RecG are unable to survive in the absence of RNase HI [33, 50]. Over-replication of the termination area has previously been reported in cells lacking RNase HI [24, 87–89], but our genetic and cell biology analyses indicate that this over-replication is not caused by a specific termination defect [24]. The effects are consistent with DNA synthesis being initiated at persistent R-loops [24], and a recent study by Raghunathan and co-workers showed that DNA synthesis initiated at various chromosomal sites can accumulate in the termination region due to the fork-blocking properties of the RFT [69].

**Figure 6.**
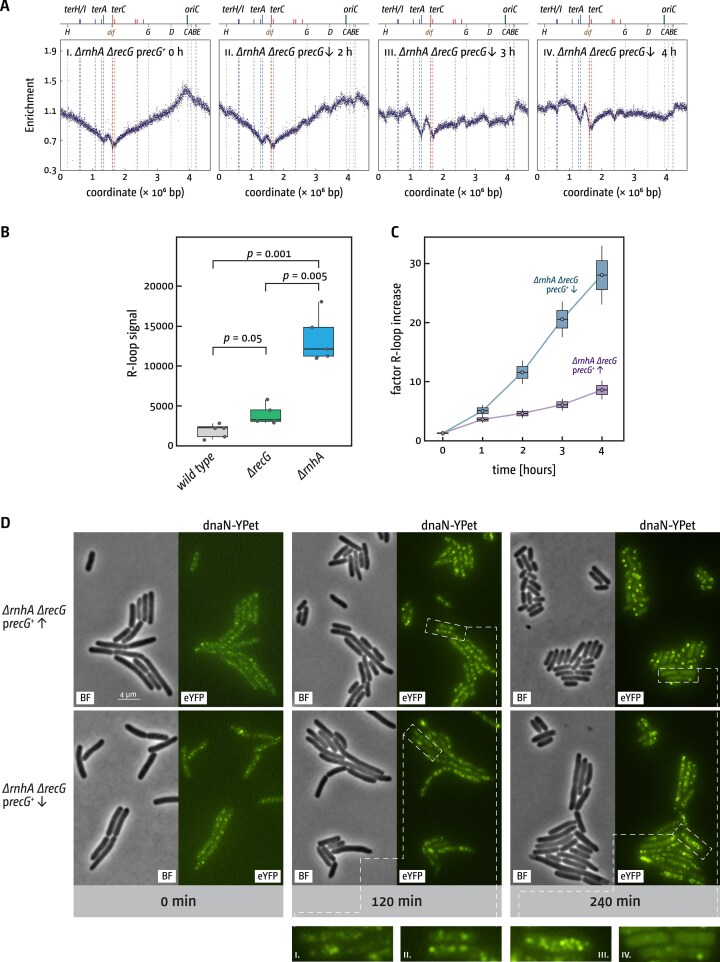
DNA synthesis, chromosome content, and R-loop levels in cells lacking RecG helicase and RNase HI. (**A**) *∆recG ∆rnhA* p*recG* cells were grown in rich medium with arabinose to maintain *recG* expression to early exponential phase. Cells were then washed and resuspended in rich medium with glucose instead of arabinose, switching the expression of the *recG* gene off (downwards pointing arrow). The number of sequence reads (normalized against reads for a stationary-phase wild-type control) is plotted against the chromosomal location. A schematic representation of the *E. coli* chromosome showing positions of *oriC* and *ter* sites (above) as well as *dif* and *rrn* operons *A–E, G*, and *H* (below) is shown above the plotted data. Sequencing templates were isolated from JD1450 (*∆rnhA ∆recG* p*recG^+^*). (**B**) Detection of R-loops in genomic DNA in the presence and absence of RecG helicase and RNase HI. Cells were grown in rich medium into early exponential phase. Genomic DNA was extracted, the concentration normalized, and equal amounts spotted onto a nitrocellulose membrane. R-loops were visualized and quantified using the R-loop-specific S9.6 antibody, followed by secondary antibody binding and induction of chemiluminescence (see the ‘Materials and methods’ section for details). The strains used were MG1655 (wild type), DG040 (*∆recG*), and AM1974 (*∆rnhA*). (**C**) Detection of R-loops in genomic DNA of cells lacking both RecG helicase and RNase HI. Cells carrying deletions of the genes for RecG helicase and RNase HI, with the deletion of *recG* covered by a plasmid with an arabinose-inducible *recG* gene, were grown in rich medium with arabinose to maintain *recG* expression to early exponential phase. Cells were then washed and resuspended in rich medium with either arabinose (purple data) or glucose (blue data), either maintaining expression of *recG* or switching it off. Samples were taken at the times indicated. Genomic DNA was extracted, the concentration normalized, and equal amounts spotted onto a nitrocellulose membrane. R-loops were visualized and quantified using the R-loop-specific S9.6 antibody, followed by secondary antibody binding and induction of chemiluminescence (see the ‘Materials and methods’ section for details). The strain used was JD1450 (*∆rnhA ∆recG* p*recG^+^*). (**D**) Visualization of DNA replication in cells lacking either RNaseHI or both RecG helicase and RNase HI via a fluorescent fusion of the β sliding clamp with the YFP derivative YPet. Cells carrying deletions of the genes for RecG helicase and RNase HI, together with an allele encoding YPet-DnaN and with the deletion of *recG* covered by a plasmid with an arabinose-inducible *recG* gene, were grown in rich medium with arabinose to maintain *recG* expression to early exponential phase. Cells were then washed and resuspended in rich medium with either arabinose (upward arrow) or glucose (downward arrow), either maintaining expression of *recG* or switching it off. Samples were taken at the times indicated. The strain used was RCe951 (*ΔrecG ΔrnhA ypet-dnaN* p*recG^+^*). For controls, see Supplementary Fig. S6.

To investigate this further, we generated replication profiles for cells lacking both RecG and RNase HI, analogous to those obtained for cells lacking RecG and 3′ exonucleases (Fig. 5). If lethality in the double mutant arises from mechanisms unrelated to termination, the replication profiles should display distinct features. Specifically, the extensive over-replication observed in the termination region of cells lacking both RecG and 3′ exonucleases should be absent. This prediction was confirmed experimentally: the increase in synthesis across the termination region was only mild (Fig. 6A).

The replication profiles also showed progressive flattening over time, which may reflect reduced ongoing synthesis as cells approach stationary phase, increased utilization of variable initiation sites, or both. A completely flat replication profile has previously been observed in *Haloferax volcanii* cells lacking all replication origins [90], consistent with replication initiation occurring at many chromosomal locations. As seen before (Fig. 5B), distinct valleys appeared at specific loci, including the *rrn* operons (Fig. 6A).

New features were also detected. Notably, the native origin exhibited diminished firing activity, while a new initiation site emerged downstream of the *rrnE* operon, consistent with previous observations in cells lacking RNase HI [24, 87–89].

To explore the possible causes of the extensive synthesis observed in the absence of both RecG and RNase HI, we considered earlier proposals that the synthetic lethality of *recG rnhA* cells [33, 50] results from excessive R-loop accumulation, which interferes with normal DNA metabolism. R-loops are known to compromise growth and viability in *E. coli* and *B. subtilis* [91] and can act as unscheduled initiation sites for DNA replication outside *oriC* [55, 92], thereby allowing cells lacking RNase HI to survive even in the absence of a functional chromosomal origin [23, 55].

To determine whether *recG rnhA* cells accumulate elevated R-loop levels, we employed the dot blot methodology previously described by Raghunathan and co-workers [69]. The S9.6 monoclonal antibody has been established as a reliable tool for detecting R-loops, three-stranded nucleic acid structures comprising an RNA:DNA hybrid and a displaced single DNA strand [52, 53]. S9.6 can theoretically bind to structures other than R-loops [93]. Therefore, we tested the specificity of the antibody signal for R-loops by treatment of genomic DNA with RNase HI, which completely eliminated the signal, while RNases A and III had substantially smaller effects (Supplementary Fig. S5A), as reported [70]. This differential response to RNase treatments demonstrates that the majority of signal detected in *E. coli* genomic DNA extracts using S9.6 represents bona fide R-loops that are substrates for RNase HI [94]. In line with this, we observed that the R-loop signal is much stronger in genomic DNA extracted from exponentially growing cultures than in stationary cultures (Supplementary Fig. S5B). One important source of R-loops is the hybridization of nascent untranslated transcripts with genomic DNA [51] and transcriptional activity of many metabolic pathways is reduced in stationary phase [95], in line with this result.

Cells lacking RNase HI exhibited a clear increase in R-loop levels, consistent with its established mechanistic role in maintaining R-loop homeostasis (Fig. 6B). In *∆recG* single mutants we found a modest, yet statistically significant, increase in R-loop signal (Fig. 6B), in contrast to the work by Raghunathan and co-workers, which did not detect an increase [69]. A role of RecG in R-loop metabolism was shown before [42, 43], both *in vitro* and *in vivo*, and the mild increase observed in our lab would be in line with these results. It has to be noted, though, that such an effect could be direct [42, 43] or indirect [36].

We then analysed R-loop levels in *∆recG ∆rnhA* cells grown in LB broth following transcriptional downregulation of the plasmid-born copy of *recG*. Under conditions where *recG* is strongly expressed, we observed a moderate increase in detected R-loops over time (Fig. 6C, purple; Supplementary Fig. S5C). However, under conditions where *recG* expression is downregulated, we saw a steady increase of the R-loop signal over time, increasing ~30-fold 4 h into the experiment (Fig. 6B, blue; Supplementary Fig. S5C). This confirms that the combined absence of RNase HI and RecG results in substantially increased R-loop levels. Given the severity of the effect observed, increased levels of R-loops may well be responsible for the observed synthetic lethality, especially as their toxic impacts were shown before [51, 52, 91]. However, importantly, this result also does not solve the question of whether RecG has a direct or indirect effect on the level of R-loops, or both.

Because R-loops can interfere with the progression of DNA replication and transcription [52], increased levels of R-loops could potentially block ongoing synthesis, explaining the flattening of the observed replication profiles (Fig. 6A). However, R-loops also serve as initiation points of DNA synthesis [55, 92]. In the absence of RNase HI processing, these persistent R-loops create multiple sites throughout the chromosome that become permissive for replisome assembly, effectively serving as distributed replication origins [24, 55, 87–89], an effect that also may explain the flattening of the replication profiles. To distinguish between these scenarios, we visualized active DNA synthesis in *∆recG ∆rnhA* cells at several time points after transcriptional downregulation of the functional copy of *recG* via the fluorescently labelled YPet-DnaN protein. In line with significant effects being visible in the replication profiles from 2 h after transcriptional downregulation, we observed a significant change in foci number and distribution in *∆recG ∆rnhA* cells in which *recG* expression was downregulated, but not in the control in which expression of *recG* was maintained (Fig. 6D; Supplementary Fig. S6). Rather than clearly defined foci, cells were filled with large numbers of ill-defined foci, forming large clusters rather than the normally observed individual foci. From 120 min into the experiment, ∼90% of cells showed these clusters, while they were observed very rarely if expression of *recG* was maintained (2 out of 280 cells showed similar clusters in the 0 control; Fig. 6D i and iii; Supplementary Figs S6 and S7A). In cells only lacking the chromosomal copy of *recG*, but with *recG* being expressed from the plasmid, foci numbers dropped towards the end of the experiment when cells entered early stationary cells. Four hours into the experiment, when transcription was maintained by the addition of arabinose, ∼45% of control cells showed no foci, whereas 4 h into the experiment, when transcription is repressed by the addition of glucose, ∼19% showed no foci. Foci clusters were not observed in these cells (Supplementary Fig. S7A).

We also analysed cell lengths in the microscopy images at the same time points. Overall, cell lengths decreased for all strains over the course of the experiment (Supplementary Fig. S7B), consistent with the transition from exponential growth towards early stationary phase [96]. In cells carrying a chromosomal deletion of *recG*, repression of plasmid-borne *recG* expression was associated with a modest increase in average cell length. This increase just reached statistical significance for the 120 min time point, while the difference for the 240 min time point was below the significance threshold. By contrast, cells lacking both *recG* and *rnhA* on the chromosome exhibited slightly larger average cell lengths and increased cell-to-cell variability. In this background, repression of plasmid-borne *recG* expression resulted in a significant shift towards larger cells (Supplementary Fig. S7B). This increase in cell lengths seems to be in line with the observed foci clusters (Figs. 6D and Supplementary Fig. S7A) and may reflect defects in chromosome segregation, activation of the SOS response by aberrant DNA intermediates, or a combination of both [97, 98].

Taken together, the observation of foci clusters in most cells lacking both RecG and RNase HI suggests that DNA synthesis is maintained in *∆recG ∆rnhA* cells at high levels throughout the time of the experiment, and it is likely that this persistent synthesis is initiated at R-loops. These extensive levels of persisting DNA synthesis are certainly in line with the observed flattening of the replication profiles (Fig. 6A). While our data preclude direct determination of the lethal mechanism in *∆recG ∆rnhA* cells, we propose that lethality results from dual R-loop-mediated pathologies: uncontrolled chromosomal over-replication and interference with essential transcriptional and replicative processes.

### Cells lacking the Tus terminator protein show increased levels of R-loops

When optimizing the experimental procedure for visualizing the level of R-loops in samples of genomic DNA, we used *∆rnhA* as a positive control [92], as well as strains where we did not expect to see any change in the level of R-loops. This included cells lacking RecG, as a previous study showed R-loop levels similar to wild-type cells [69], and cells lacking Tus, which has DNA binding but no R-loop processing activity [9, 99].

**Figure 7.**
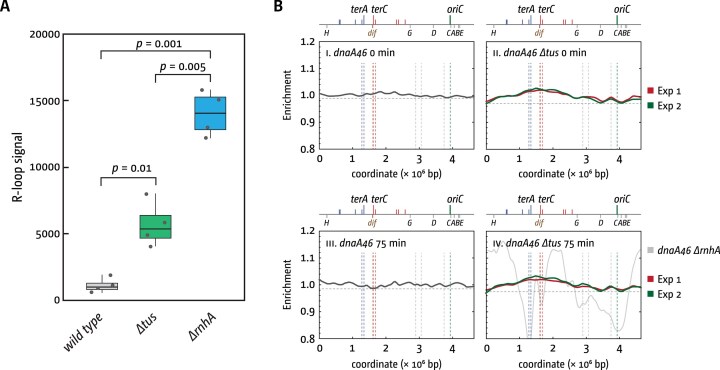
R-loop levels and replication profiles in the presence or absence of Tus terminator protein. (**A**) Cells were grown in rich medium into early exponential phase. Genomic DNA was extracted, the concentration normalized, and equal amounts spotted onto a nitrocellulose membrane. R-loops were visualized and quantified using the R-loop-specific S9.6 antibody, followed by secondary antibody binding and induction of chemiluminescence (see the ‘Materials and methods’ section for details). The strains used were MG1655, AM1775 (*∆tus*), and AM1974 (*∆rnhA*). (**B**) LOESS regression of replication profiles of synchronized *dnaA46* cultures in the presence and absence of *tus*. For cells lacking Tus LOESS regressions from data from two independent biological replicates (in red and green) are shown to highlight common features (shown by grey dashed lines in all panels). A LOESS regression profile for *dnaA46 ∆rnhA* cells is shown for comparison in grey in panel iv; the profile was replotted from data published previously [24]. Cells were grown to early logarithmic phase at permissive temperature of 30°C and then shifted to restrictive temperature of 42°C for 90 min to let all ongoing rounds of DNA replication finish while initiation of new rounds of synthesis at *oriC* is inhibited. At this point the samples for the data shown in panels (i and ii) were taken. For panels (iii and iv), the cultures were shifted to permissive temperature for 30 min to allow one round to be initiated at *oriC*. Cultures were then shifted back to restrictive temperature for 45 more minutes to inhibit any further rounds of synthesis from being initiated. For the full replication profiles, see Supplementary Fig. S8. The strains used were AU1054 (*dnaA46*) and RCe203 (*dnaA46 ∆tus*).

We found, unexpectedly, that the R-loop signal was increased in *∆tus* single mutants (Fig. 7A). Tus is a 308-amino-acid protein (35.6 kDa) that binds tightly to *ter* sites such as *terB* through a DNA-binding cleft, but it has no reported functions related to R-loop metabolism [9, 100–102]. To confirm that the increase was specific and reproducible, we performed several validation experiments. First, RNase HI pre-treatment of genomic DNA abolished the signal, confirming its R-loop specificity (Supplementary Fig. S8A). Second, to rule out strain-specific artefacts, we backcrossed the *∆tus::cat* allele into the MG1655 background and verified the deletion by PCR (Supplementary Fig. S8B and C). The backcrossed strain reproduced the elevated R-loop phenotype (Supplementary Fig. S8D). Finally, an independently generated *tus1::dhfr* allele, obtained via a distinct mutagenesis strategy, exhibited the same R-loop accumulation (Supplementary Fig. S8D). The consistent results from these two independent *∆tus* alleles strongly support that the increased R-loop signal is indeed a consequence of Tus deficiency.

Where are persisting R-loops in the absence of the Tus–*ter* system coming from? The mechanism underlying elevated R-loop levels in Tus-deficient cells remains unclear. This phenomenon may reflect either localized R-loop formation hotspots or global increases in R-loop accumulation. The latter could result from transcriptional upregulation if Tus normally functions to repress expression of specific gene sets. However, with the exception of Tus regulating its own expression, with *terB* being located within the promoter region of *tus* [103], there is no indication that Tus is a transcriptional regulator of other genes. Previous replication profiling studies of Tus-deficient strains [23, 24, 38, 39] revealed profiles largely resembling wild type, with only subtle deviations observed in the termination area. We observed no evidence of R-loop hotspots disrupting DNA replication [104] or ectopic initiation sites operating independently of *oriC*. If the R-loops that persist in the absence of Tus can trigger DNA synthesis, similar to the R-loops persisting in cells lacking RNase HI, then we suspect that there are no distinct R-loop hotspots. Instead, R-loops might occur in a wider variety of locations, which, in this type of bulk analysis, would show a dispersed low-level signal over a larger chromosomal area.

To get some insight into whether low levels of DNA synthesis might be observed if background noise is reduced, we synchronized DNA replication in cells with and without Tus via the temperature-sensitive *dnaA46* allele. Following growth to early exponential phase at permissive temperature (30°C), we shifted cultures to restrictive temperature (42°C) for 90 min, which allowed all ongoing rounds of synthesis to finish while firing of *oriC* is inhibited. We then shifted cells to permissive temperature, but only for 30 min before shifting cultures back to restrictive temperature, to allow initiation of a single round of DNA synthesis without further rounds being established.

Both *dnaA46* single mutants and *dnaA46 ∆tus* double mutants exhibited a distinct temporal progression, transitioning from initially flat profiles through intermediate stages reflecting active DNA synthesis, ultimately returning to flat profiles upon replication completion (Supplementary Fig. S9). Overall, the profiles of *dnaA46 ∆tus* cells were very similar. The profiles showed no discrete features indicative of replication fork impediment and no defined chromosomal locations where origin-independent synthesis was initiated (Supplementary Fig. S9). However, comparison of the flat profiles at the beginning and end of the time course revealed localized increases in marker frequency in the absence of Tus. We have simplified the profiles in Fig. 7B by only showing the LOESS regression analysis of the profiles of *dnaA46* and *dnaA46 ∆tus* cells at 0 and 75 min (see Supplementary Fig. S9 for a complete set of profile data). The largest of these increases is almost 3 Mbp in width and spans the entire termination domain of the chromosome (Fig. 7B ii and iv), in line with our previous profiles of unsynchronized cells, which showed effects in the termination area [11, 23]. What appears to be over-replication fits precisely with what was observed before: a mild level of over-replication in cells lacking Tus [31]. It could be entirely explained by a low level of over-replication triggered at fork fusion points (Fig. 1). However, based on the observation that higher levels of R-loops are observed, it is entirely possible that initiation of DNA synthesis is responsible, or a combination of both factors. In addition, the comparison of the data from two sequencing runs (shown in red and green in Fig. 7B ii and iv) shows at least two additional and more defined peaks, one around 3.7 Mbp and one peak, or perhaps two merging peaks, at around 3 Mbp (locations highlighted by grey dashed lines in all panels of Fig. 7B). If these locations are indeed R-loops triggering DNA synthesis, then it highlights again that the effects of Tus inactivation are rather global, as all of them are closer to the origin than the termination area and certainly far away from any *ter* sites. However, it is noteworthy that the effects can only be visualized if the profiles are significantly magnified. To highlight the difference in scale, we have plotted a LOESS regression analysis from a raw data set from *dnaA46 ∆rnhA* cells grown at 42°C published previously [24] in panel iv of Fig. 7B, showing the height of peaks in cells in which synthesis can be retained without origin firing [24]. The comparison of these data sets highlights the relatively mild effects observed in cells lacking Tus. Notably, the observed increase in R-loops in *∆tus* cells is unlikely to arise from indirect depletion of R-loop–processing enzymes, such as RecG and RNase HI. Both act in mechanistically distinct pathways and cannot compensate for one another *in vivo* [24], and loss of Tus does not produce phenotypes resembling RecG deficiency.

Regardless of where and how precisely the observed elevated R-loop levels are formed, interference of R-loops with DNA replication is a source of genomic instability [105], and our data from cells lacking RecG and RNase HI are in line with increased levels of R-loops being associated with lethality of cells affected (Fig. 6D), as shown before [91]. These findings indicate that RFT systems may facilitate R-loop homeostasis, suggesting that termination of DNA replication, either directly or indirectly, can promote R-loop formation when not spatially constrained by a fork trap mechanism. Further investigations are required to elucidate the underlying molecular mechanisms. Nevertheless, our findings demonstrate that replication termination destabilizes chromosomes through at least two pathways: localized instability at fork fusion sites and global R-loop accumulation throughout unspecified chromosomal regions. These results underscore the importance of fork trap-mediated containment systems, particularly given that such pathologies become severely amplified when essential processing factors like RecG helicase or 3′ exonucleases are absent.

## Discussion

RFTs were first identified in *E. coli* in the late 1970s and shortly thereafter in *B. subtilis* [18, 106, 107]. Although their biochemical properties are well characterized, their role *in vivo* remains less clear. Fork trap systems are present in only a subset of bacterial lineages, including the Enterobacteriales, *Pseudoalteromonas*, and most Aeromonadales [15], suggesting that the majority of bacterial species successfully manage fork fusion events without them. The phenotypes of *∆tus* cells described so far are relatively modest [16, 31, 108, 109]. This makes the strict conservation of the fork trap across all *E. coli* phylogroups and related species, together with its independent emergence in *B. subtilis*, seem paradoxical [9, 15, 16, 21]. Its maintenance likely reflects the severity of fork fusion-associated pathologies, including chromosomal over-replication, localized genomic instability, and increased R-loop accumulation, which become more pronounced when processing pathways are compromised.

Our study supports four main conclusions. (1) Fork fusion events directly induce local increases in recombination within chromosomal regions undergoing termination. (2) Concurrent loss of factors that process fork fusion intermediates, such as RecG helicase and 3′ exonucleases, leads to pronounced over-replication in the termination area, underscoring the severity of unprocessed fork fusion structures. (3) Cells lacking both RecG and RNase HI accumulate high levels of R-loops. Rather than generating localized termination defects, these R-loops drive widespread over-replication across the entire chromosome, ultimately leading to lethality. (4) The RFT influences R-loop metabolism: *∆tus* cells exhibit elevated R-loop levels that appear globally distributed rather than restricted to defined loci. Our findings advance our understanding of how termination contributes to genome stability and how defects in fusion processing intersect with R-loop biology. They also help explain both the exclusive use of single replication origins in bacteria and the selective advantage conferred by RFT systems.

Bacterial chromosomes are normally replicated from a single *oriC*, where two bidirectional forks are loaded, which eventually meet in the terminus region [1]. This contrasts sharply with archaea and eukaryotes, where chromosomes are replicated from multiple origins [2, 110]. The exclusive use of a single origin is surprising because it creates a potential bottleneck: successful cell division depends on the rate of DNA synthesis, which in *E. coli* reaches 550–750 bp/s [111]. This is almost 20-fold faster than the speed of synthesis in human cells [112]. While multiple origins may be needed for timely duplication of large eukaryotic genomes, even archaeal chromosomes of bacterial size often employ several origins [110, 113]. Bacteria accelerate their cell cycle through overlapping rounds of replication, in which new initiation events occur before previous rounds have completed [114]. The use of multiple origins would be another simple solution to this problem, especially as bacteria can tolerate the presence of multiple origins without major ill-effects, at least in some locations [15, 38, 39, 79, 115]. Nevertheless, no bacterial species has been found that naturally replicates its chromosome from more than one origin under normal growth conditions.

Multiple factors likely contributed to the evolutionary adoption of single replication origins in bacteria, including the ability to control chromosome duplication precisely and avoid over-replication [116]. However, work from several groups has shown that termination events themselves can undermine this control: fork fusions can generate substantial over-replication at fusion sites [23, 25, 31]. Our results extend this view by demonstrating that recombination frequencies increase specifically at sites where forks fuse (Figs 2 and 3), even in cells with fully functional repair pathways, indicating a direct link between fork fusion and local genomic instability. Our data further suggest that the RFT contributes to maintaining global R-loop homeostasis through an as-yet unknown mechanism.

**Figure 8.**
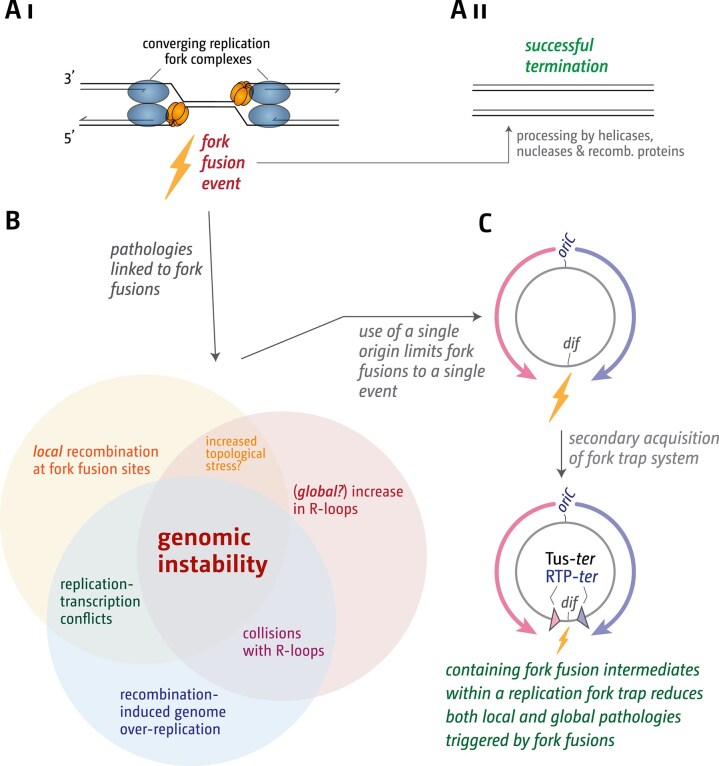
Two replication fork complexes must fuse when DNA replication terminates. (**A**) Fork fusions (A i) need to be carefully managed in bacterial cells to achieve successful termination (A ii). (**B**) If not adequately processed, fork fusions result in a wide variety of issues that result in genomic instability, as highlighted by the Venn diagram. (**C**) We hypothesize that the negative effects shown in panel (B) have shaped the architecture of bacterial chromosomes. See text for further details.

Although genomic instability can facilitate adaptation in fluctuating environments, it is generally costly in stable conditions or near a fitness peak [117]. If fork fusions threaten replication control, promote local instability, and alter R-loop equilibrium (Fig. 8), these consequences could impose selective pressure favouring genomes that minimize such events. This framework helps explain why later acquisition of an RFT could be advantageous and why, once established, it is consistently maintained. Fork trap systems efficiently confine mild to moderate over-replication triggered by fork fusion [23, 25, 27], allowing faster processing and resolution of intermediates and limiting downstream consequences such as recombination-driven synthesis. This might well explain our data, which suggest that an active fork trap mechanism reduces recombination frequencies in locations where forks fuse (Fig. 4), likely by containing downstream consequences such as replication triggered at recombination intermediates.

Our finding that the fork trap influences R-loop homeostasis further underscores the role of genomic instability in shaping termination mechanisms (Fig. 7). R-loops can initiate over-replication at multiple chromosomal sites, enabling *∆rnhA* cells to replicate without origin firing [24, 55, 87, 88]. Their persistence therefore threatens the precise control of chromosome duplication [116]. Unexpectedly, loss of Tus alone caused a detectable increase in R-loops (Fig. 7), even though Tus is solely a DNA-binding protein with no known role in R-loop metabolism [9]. This suggests an indirect effect, potentially by altering conditions that favour R-loop formation or persistence.

We detected only few specific chromosomal hotspots of R-loop–driven synthesis in *∆tus* cells (Fig. 7 and Supplementary Fig. S8). Instead, we observed a broad peak that covered the entire termination domain of the chromosome. This implies that R-loop persistence increases broadly rather than at defined loci, and even if defined loci exist, these are distal from the termination area (Fig. 7). The moderate over-replication previously observed in *∆tus* strains [31] may therefore arise from fork fusion events, R-loops, or a combination of both. Regardless of the precise mechanism, the fork trap appears to limit the accumulation of R-loops and thereby constrains both R-loop–triggered over-replication and R-loop–mediated interference with replication and transcription (Fig. 8).

The data presented here and elsewhere support the idea that fork trap systems reduce the impact of several fork fusion-mediated pathologies, explaining why their acquisition from a plasmid precursor was advantageous [15] and why, once present, they are consistently maintained (Fig. 8) [9, 16]. At the same time, the proteins that process fusion intermediates are widely conserved across bacteria, indicating that any species lacking a fork trap still possess the repair machinery needed to manage termination-associated problems. Fork traps are therefore not essential for viability but instead provide an additional containment mechanism that spatially restricts problematic intermediates and facilitates their efficient resolution.

In cells lacking multiple key termination proteins, such as RecG and 3′ exonucleases, over-replication in the termination area is increased to such an extent that it is not fully contained by the RFT (Fig. 5), an effect observed before in cells with high levels of over-replication [23, 25], [27]. The synthetic lethality of *∆recG* cells lacking all 3′ exonuclease activities [33] has been interpreted as evidence that excessive over-replication and recombination overwhelm remaining repair pathways. The data presented here highlight that this view may be too simplistic. There is no doubt that extensive levels of over-replication can be problematic. We showed before that cells that carried an additional extra replication origin showed very high levels of over-replication within the termination area in the absence of RecG helicase [23, 25]. Furthermore, *∆recG* cells with only an ectopic replication origin were synthetically lethal, an effect suppressed by the inactivation of the Tus–*ter* fork trap system [25]. These results strongly suggest that cells struggle with high levels of fork fusion-induced over-replication, especially if forks get trapped within the fork trap area. The precise reason for the lethality is unclear, and several mechanisms might contribute, including recombination at double-stranded DNA ends generated by forks stemming from over-replication running into forks arrested at Tus–*ter* complexes [78, 118].

But our results indicate that the situation is more complex than previously appreciated. Despite the extreme over-replication observed in the terminus region, *∆recG* cells lacking one or two 3′ exonucleases do not exhibit major growth defects, showing that even synthesis exceeding that from *oriC* can be tolerated (Fig. 5). In contrast, *∆recG* cells lacking all three major 3′ exonucleases display pronounced distortions in their replication profiles at multiple chromosomal obstacles, including the *rrn* operons and some Tus–*ter* complexes (Fig. 5). Similar defects arise in *∆recG ∆rnhA* cells, where lethality is likely driven by excessive R-loops and R-loop-initiated synthesis (Fig. 6).

Notably, *∆recG ∆rnhA* strains showed strong distortions at *terH* and *terI*, sites where Tus binding is normally undetectable *in vivo* [10]. Although altered binding cannot be excluded, we favour the interpretation that impaired fork-processing capacity reveals weak Tus barriers that are efficiently removed in wild-type cells but become problematic when fork removal mechanisms are compromised (Fig. 6). Whether lethality results from these obstacles alone or from their combination with pervasive over-replication remains to be established. Further work will be required to define how termination-associated pathologies influence fork progression, genomic stability, transcription, and R-loop dynamics and how these pressures shape bacterial chromosome evolution.

Given the instability triggered by even a single fork fusion in bacteria, it remains striking that eukaryotic cells routinely complete hundreds to thousands of termination events without catastrophic consequences. Termination likely presents different challenges in eukaryotes. In *E. coli*, genetic and biochemical data identify a 3′ flap, which is an inherently recombinogenic structure, as a key intermediate underlying several termination-associated pathologies [4, 23, 24, 26, 32, 119]. Our working model predicts that this intermediate is generated by the interaction of the DnaB helicase of one fork with the nascent leading strand of the opposing fork (Fig. 1). As noted before [84], the eukaryotic MCM helicase has the opposite polarity to DnaB in bacteria [120]. A similar encounter at fusing forks, if it were to happen, would therefore generate a 5′ flap structure, which is not recombinogenic. 5′ flaps are common intermediates in Okazaki fragment processing and therefore rapidly processed by proteins such as FEN1 and Dna2 nucleases and Pif1 helicase [121, 122]. Nevertheless, studies of termination in eukaryotic cells confirm that this stage of chromosome duplication is highly complex and that a variety of factors are needed to bring replication to a successful conclusion [5, 6, 123–127]. Considerable gaps remain in our understanding of this process, underscoring the need for further investigations of this complex and fascinating stage of the chromosome duplication process.

## Supplementary Material

gkaf1519_Supplemental_File

## Data Availability

Raw sequencing data can be accessed at the NIH Sequence Read Archive under accession number PRJNA1244804.
