# Supplementary material for: Termination of DNA replication drives genomic instability via multiple mechanisms
Source: Nucleic Acids Res. 2026 Jan 16;54(2):gkaf1519. doi: 10.1093/nar/gkaf1519 (PMC12809603; doi:10.1093/nar/gkaf1519)
Supplement: gkaf1519_Supplemental_File [file gkaf1519_supplemental_file.pdf]

Supplementary Material

for:

Termination of DNA replication drives genomic instability via multiple mechanisms

Daniel J. Goodall[1], Juachi U. Dimude[1], M. Amin Hashemloo[1], Emma L. Dunbar[2], Iren Grigoryan[1], Amy L. Upton[1], Edward L. Bolt[3] and Christian J. Rudolph[1,*]

*Corresponding author: christian.rudolph@brunel.ac.uk

1 Division of Biosciences, College of Health, Medicine and Life Sciences, Brunel University of London, Uxbridge, UK

2 Department of Biochemistry, University of Wisconsin-Madison, Madison, USA

3 School of Life Sciences, University of Nottingham, UK

SUPPLEMENTARY TABLES

Table S1. *Escherichia coli* K12 strains

Strain number	Relevant Genotype[a]	Source
**MG1655 derivatives**		
MG1655	F^- *rph-1*	(1)
AM1655	*ΔrecG::apra*	(2)
AM1666	*ΔrecA::apra*	(3)
AM1775	*Δtus::cat*	(4)
AM1874	*ΔxseA::dhfr*	(2)
AM1974	*ΔrnhA::dhfr*	A.A. Mahdi & R.G. Lloyd, unpublished
AS1062	*<kan>-ypet-dnaN*	(5)
AU1014	*priA300 ΔlacIZYA ΔrecG::apra*	White colony of JJ1078
AU1015	*ΔlacIZYA ΔrecG::apra*	(6)
AU1020	*ΔlacIZYA ΔrnhA::cat* pJJ100	N6283 × P1.N4704 to Cm^r
AU1054	*tnaA::*Tn*10 dnaA46*	(4)
AU1185	*ΔrecG263::kan* pDIM104	N4256 × pDIM104 to Ap^r
AU1188	*ΔrecG263::kan ΔrnhA::cat* pDIM104[b]	AU1185 × P1.N4704 to Cm^r Ap^r
DG010	*kankanMX4-<cat>-yjhR*[c]	This study
DG011	*kankanMX4-<cat>-narU tus1::dhfr*	SLM1042 × P1.N6798 to Tm^r
DG024	*ΔlacIZYA pheA::oriX-<> kankanMX4-<cat>-yjhR*	JD1338 × P1.DG010 to Cm^r
DG026	*ΔlacIZYA oriZ-<> kankanMX4-<cat>-yjhR*	JD1339 × P1.DG010 to Cm^r
DG028	*ΔlacIZYA oriZ-<> kankanMX4-<cat>-narU*	JD1339 × P1.SLM1042 to Cm^r
DG033	*ΔlacIZYA oriZ-<> kankanMX4-<cat>-narU tus1::dhfr*	DG028 × P1.DG011 to Tm^r
DG040	*ΔrecG::apra*	MG1655 × P1.AU1014 to $Apra^r$
DG059	*kankanMX4-<cat>-tldD*[c]	This study
DG061	*ΔlacIZYA pheA::oriX-<> kankanMX4-<cat>-tldD*	JD1338 × P1.DG059 to Cm^r
DG062	*ΔlacIZYA oriZ-<> kankanMX4-<cat>-tldD*	JD1339 × P1.DG059 to Cm^r
DG069	*ΔlacIZYA oriZ-<> kankanMX4-<cat>-yjhR ΔrecG::apra*	DG026 × P1.DG040 to $Apra^r$
DG076	*kankanMX4-<cat>-yjhR*[c] *ΔrecA::apra*	DG010 × P1.AM1666 to $Apra^r$
DG077	*ΔlacIZYA oriZ-<> kankanMX4-<cat>-yjhR ΔrecA::apra*	DG026 × P1. AM1666 to $Apra^r$
DL729	*ΔsbcCD::kan recD1009 supE supF*	David Leach
JD1017	*ΔxonA::apra ΔsbcCD::kan*	RCe563 × P1.N5296 to Km^r
JD1041	*ΔlacIZYA ΔrnhA::cat*	White colony from AU1020
JD1072	*ΔlacIZYA ΔrnhA::cat* pAM490	JD1041 × pAM490 to Ap^r
JD1181	*ΔlacIZYA pheA::oriX-<cat>*	(7)
JD1338	*ΔlacIZYA pheA::oriX-<>*	JD1181 × pCP20 to Cm^s Ap^s
JD1339	*ΔlacIZYA oriZ-<>*	(7)
JD1443	*ΔlacIZYA ΔrnhA::cat* pDIM104[b]	JD1041 × pDIM104 to Ap^r
JD1450	*ΔlacIZYA ΔrnhA::cat ΔrecG::apra* pDIM104[b]	JD1443 × P1.AU1015 to $Apra^r$
JD1504	*ΔxseA::dhfr ΔxonA::apra ΔsbcCD::kan*	SLM1203 × P1.RCe562 to Km^r
JD1514	*ΔxseA::dhfr ΔsbcCD::kan* pDIM104[b]	SLM1209 × pDIM104 to Ap^r
JD1516	*ΔxseA::dhfr ΔsbcCD::kan ΔrecG265::cat* pDIM104[b]	JD1514 × P1.N4560 to Cm^r Ap^r
JD1521	*ΔxseA::dhfr ΔsbcCD::kan ΔrecG265::cat ΔxonA::apra* pDIM104[b]	JD1516 × P1.RCe563 to $Apra^r$ Ap^r
JD1576	*ΔxseA::dhfr ΔsbcCD::kan ΔrecG265::cat*	SLM1209 × P1.N4560 to Cm^r
JD1577	*ΔxonA::apra ΔsbcCD::kan ΔrecG265::cat*	RCe569 × P1.N4560 to Cm^r
JD1579	*ΔxseA::dhfr ΔxonA::apra ΔrecG265::cat*	SLM1203 × P1.N4560 to Cm^r
JD1587	*ΔxseA::dhfr ΔrecG::apra*	AM1874 × P1.AU1015 to $Apra^r$

JJ1060	*priA300 ΔlacIZYA*	White colony of N5933
JJ1075	*priA300 ΔlacIZYA ΔrecG::apra*	JJ1060 × P1.AM1655 to Apra^r
JJ1078	*priA300 ΔlacIZYA ΔrecG::apra* pJJ100	JJ1075 × pJJ100 to Ap^r
JJ1359	*ΔlacIZYA dam1::kan ΔrecG::apra tus1::dhfr*	J. Zhang and R.G. Lloyd, unpublished
N4256	*ΔrecG263::kan*	(8)
N4560	*ΔrecG265::cat*	(9)
N4704	*ΔrnhA::cat*	(2)
N5286	*xonAΔ300::cat*	MG1655 × P1.N5005 to Cm^r
N5296	*xonAΔ300::cat ΔsbcCD::kan*	N5286 × P1.DL729 to Km^r
N5933	*priA300 ΔlacIZYA* pAM374	(3)
N6283	*ΔlacIZYA* pJJ100	(2)
N6798	*ΔrecG265::cat tus1::dhfr*	(7)
N6798	*ΔrecG265::cat tus1::dhfr*	N4560 × P1.JJ1359 to Tm^r
RCe203	*tnaA::*Tn*10 dnaA46 Δtus::kan*	(4)
RCe504	*oriZ-cat-frt*	(10)
RCe562	*ΔsbcCD::kan*	(11)
RCe563	*ΔxonA::apra*	(11)
RCe567	*oriZ-cat-frt tus1::dhfr*	(10)
RCe569	*ΔxonA::apra ΔsbcCD::kan*	(11)
RCe632	*ΔlacIZYA ΔrnhA::cat tus1::dhfr* pAM490	JD1072 × P1.N6798 to Tm^r Ap^r
RCe633	*ΔlacIZYA ΔrnhA::cat tus1::dhfr*	Plasmid-free derivative of RCe632
RCe666	*ΔsbcCD::kan ΔrecG265::cat*	RCe562 × P1.N4560 to Cm^r
RCe667	*ΔxonA::apra ΔrecG265::cat*	RCe563 × P1.N4560 to Cm^r
RCe749	*oriZ-cat-frt <kan>-ypet-dnaN*	RCe504 × P1.AS1062 to Km^r
RCe759	*oriZ-cat-frt tus1::dhfr <kan>-ypet-dnaN*	RCe567 × P1.AS1062 to Km^r
RCe766	*ΔrecG::apra <kan>-ypet-dnaN*	AM1655 × P1.AS1062 to Km^r
RCe941	*ΔrecG::apra <kan>-ypet-dnaN* pDIM104[b]	RCe766 × pDIM104 to Ap^r
RCe942	*tus1::dhfr*	MG1655 × P1.RCe759 to Tm^r
RCe951	*ΔrecG::apra <kan>-ypet-dnaN ΔrnhA::cat* pDIM104[b]	RCe941 × RCe633 to Cm^r Ap^r
RCe960	*Δtus::cat*	MG1655 × P1.AM1775 to Cm^r
SLM1037	*kankanMX4-<cat>-narU*[c]	This study
SLM1042	*kankanMX4-<cat>-narU*	MG1655 × P1.SLM1037 to Cm^r
SLM1185	*ΔxseA::dhfr*	(11)
SLM1203	*ΔxseA::dhfr ΔxonA::apra*	(11)
SLM1209	*ΔxseA::dhfr ΔsbcCD::kan*	(11)
STL2694	*xonAΔ300::cat thr-1 leuB6 proA2 supE44 kdg51 rfbD1 araC14 lacY1 galK2 xyl-5 mtl-1 tsx-33 rpsL31 rac*$^-$	Susan Lovett

a – Only the relevant additional genotype of the derivatives is shown. The abbreviations 'kan', 'apra', 'dhfr' and 'cat' refer to insertions conferring resistance kanamycin (Km^r), apramycin (Apra^r), trimethoprim (Tm^r) and chloramphenicol (Cm^r). '<>' indicates the use of *frt* sites, where *frt* stands for the 34 bp recognition site of the FLP/*frt* site-directed recombination system. Thus, *<cat>* refers to a chloramphenicol marker flanked by an *frt* site either side. If the *frt* site was removed via Flp recombinase, this is shown by '<>', indicating that a single *frt* scar is left in the chromosome.

b – All plasmids shown carry an ampicillin resistance marker (Ap^r). pDIM104 carries the *recG* coding sequence, cloned behind the P_{araBAD} promoter of pLAU17 (12) using the NcoI and XbaI sites, thus replacing eCFP (13).

c – For a description of the chromosomal integrations of the recombination reporter cassette please see Material & Methods.

SUPPLEMENTARY REFERENCES

1. Bachmann, B J (1996) Derivations and Genotypes of Some Mutant Derivatives of Escherichia coli K-12. In *Escherichia coli and Salmonella Cellular and Molecular Biology*. ASM Press.

2. Rudolph,C.J., Mahdi,A.A., Upton,A.L. and Lloyd,R.G. (2010) RecG protein and single-strand DNA exonucleases avoid cell lethality associated with PriA helicase activity in Escherichia coli. *Genetics*, **186**, 473–492.

3. Mahdi,A.A., Buckman,C., Harris,L. and Lloyd,R.G. (2006) Rep and PriA helicase activities prevent RecA from provoking unnecessary recombination during replication fork repair. *Genes Dev.*, **20**, 2135–2147.

4. Rudolph,C.J., Upton,A.L., Stockum,A., Nieduszynski,C.A. and Lloyd,R.G. (2013) Avoiding chromosome pathology when replication forks collide. *Nature*, **500**, 608–611.

5. de Dios,R., Gadar,K., Proctor,C.R., Maslova,E., Han,J., Soliman,M.A.N., Krawiel,D., Dunbar,E.L., Singh,B., Peros,S., *et al.* (2025) Saccharin disrupts bacterial cell envelope stability and interferes with DNA replication dynamics. *EMBO Mol. Med.*, 10.1038/s44321-025-00219-1.

6. Midgley-Smith,S.L., Dimude,J.U., Taylor,T., Forrester,N.M., Upton,A.L., Lloyd,R.G. and Rudolph,C.J. (2018) Chromosomal over-replication in Escherichia coli recG cells is triggered by replication fork fusion and amplified if replichore symmetry is disturbed. *Nucleic Acids Res.*, **46**, 7701–7715.

7. Dimude,J.U., Stein,M., Andrzejewska,E.E., Khalifa,M.S., Gajdosova,A., Retkute,R., Skovgaard,O. and Rudolph,C.J. (2018) Origins Left, Right, and Centre: Increasing the Number of Initiation Sites in the Escherichia coli Chromosome. *Genes*, **9**.

8. Jaktaji,R.P. and Lloyd,R.G. (2003) PriA supports two distinct pathways for replication restart in UV-irradiated Escherichia coli cells. *Mol. Microbiol.*, **47**, 1091–1100.

9. Meddows,T.R., Savory,A.P. and Lloyd,R.G. (2004) RecG helicase promotes DNA double-strand break repair. *Mol. Microbiol.*, **52**, 119–132.

10. Ivanova,D., Taylor,T., Smith,S.L., Dimude,J.U., Upton,A.L., Mehrjouy,M.M., Skovgaard,O., Sherratt,D.J., Retkute,R. and Rudolph,C.J. (2015) Shaping the landscape of the Escherichia coli chromosome: replication-transcription encounters in cells with an ectopic replication origin. *Nucleic Acids Res.*, **43**, 7865–7877.

11. Midgley-Smith,S.L., Dimude,J.U. and Rudolph,C.J. (2019) A role for 3′ exonucleases at the final stages of chromosome duplication in Escherichia coli. *Nucleic Acids Res.*, **47**, 1847–1860.

12. Lau,I.F., Filipe,S.R., Søballe,B., Økstad,O.-A., Barre,F.-X. and Sherratt,D.J. (2003) Spatial and temporal organization of replicating Escherichia coli chromosomes. *Mol. Microbiol.*, **49**, 731–743.

13. Rudolph,C.J., Upton,A.L., Harris,L. and Lloyd,R.G. (2009) Pathological replication in cells lacking RecG DNA translocase. *Mol. Microbiol.*, **73**, 352–366.

14. Wach,A., Brachat,A., Pöhlmann,R. and Philippsen,P. (1994) New heterologous modules for classical or PCR-based gene disruptions in Saccharomyces cerevisiae. *Yeast Chichester Engl.*, **10**, 1793–1808.

15. Ede,C., Rudolph,C.J., Lehmann,S., Schürer,K.A. and Kramer,W. (2011) Budding yeast Mph1 promotes sister chromatid interactions by a mechanism involving strand invasion. *DNA Repair*, **10**, 45–55.

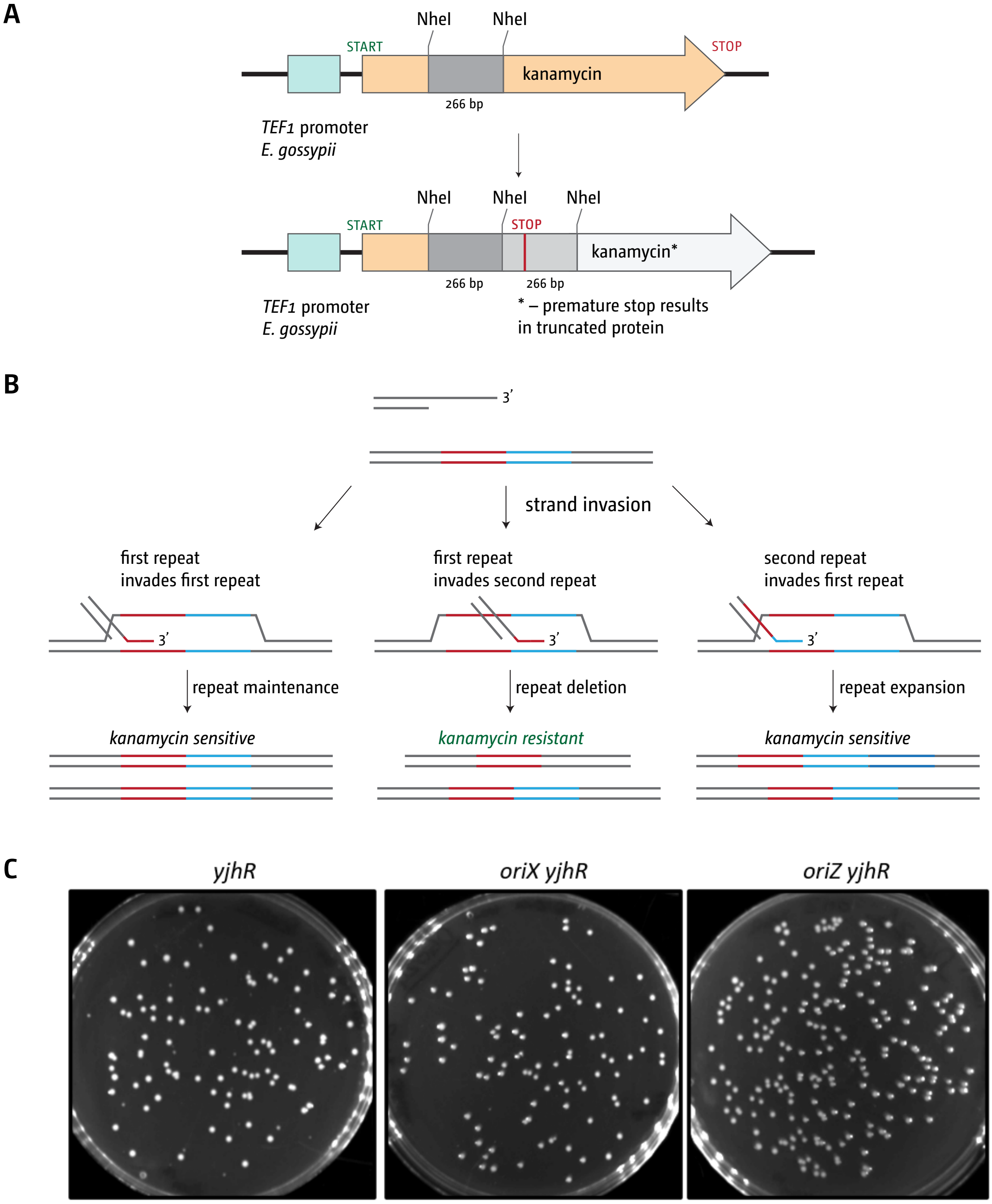

Figure S1: Fork fusion events in an ectopic location trigger increased recombination.
A) Schematic of the tandem-repeat recombination reporter cassette used. The construct is based on the kanamycin resistance marker from plasmid pFA6a-kanMX4 (14). To generate the KanKanMX4 module, a 266 bp internal NsiI fragment of the KanMX4 cassette was cloned into pFA6a-kanMX4 partially digested with NsiI, creating an internal 266 bp direct repeat. This duplication introduces a frameshift and premature stop codon, rendering the resistance gene inactive. The module was subcloned into pRS316 via EcoRI and SalI, and the cassette was sequence-verified (15). Additional modifications required for chromosomal integration are described in Materials & Methods. **B)** Schematic of possible recombination outcomes of the reporter cassette. Recombination between the repeats can maintain the tandem duplication, delete one repeat, or expand the duplication, depending on which repeated segment the double-stranded DNA end aligns with. **C)** Representative example of reversion frequencies in cells with a single origin (*oriC*$^{+}$), a second origin in the left-hand replichore (*oriC*$^{+}$ *oriX*$^{+}$) and a second origin in the right-hand replichore (*oriC*$^{+}$ *oriZ*$^{+}$). All had the kankanMX4 module integrated near the *yjhR* gene (see also main text for further details). Overnight cultures of the *E. coli* strains of interest were diluted 1:100 into 1 mL of LB (Miller) medium in 2 mL reaction tubes (Sarstedt) to achieve an initial A_{600} of 0.04. For each strain, 11 parallel cultures were grown at 37 °C in a Thermomixer (Eppendorf) with shaking at 1000 rpm to an A_{600} of 0.4. One additional culture was grown alongside the fluctuation test cultures to allow determination of the cell density via A_{600} measurement. Viable titres were determined by spotting serial dilutions of this parallel A_{600} culture onto agar plates three times and the average colony count was used to represent the number of colonies for that dilution. Dilutions of 1×10^{-5} and 1×10^{-6} were used to avoid resolution issues for higher dilutions. When the target A_{600} was reached, cultures were centrifuged, resuspended in 100 µL LB (Miller) broth and plated onto LB (Miller) agar supplemented with 40 µg/mL kanamycin. Plates were incubated at 37 °C for 24 hours and all colonies per plate counted.

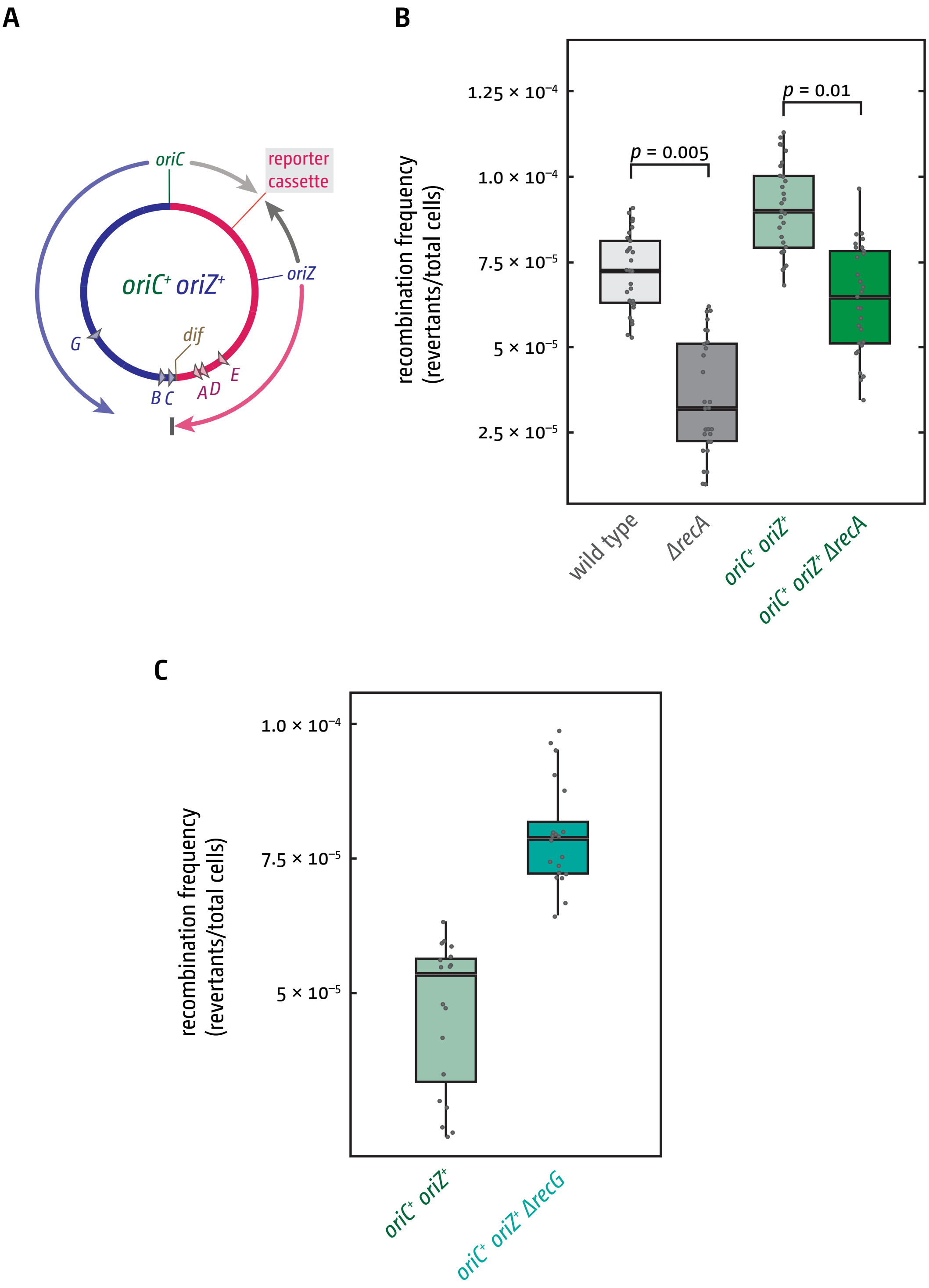

Figure S2: Effects of the presence or absence of RecA recombinase and RecG helicase on recombination events in an ectopic termination location.

A) Overview of the *E. coli* chromosome, highlighting the integration site of the tandem repeat reporter cassette at 4.53 Mbp, integration sites of the additional ectopic replication origin *oriZ*, as well as the resulting replichore configuration and termination sites. Due to the shorter replichore lengths resulting from ectopic origin integration, the fork initiating from the ectopic origin and travelling in the normal direction always reaches the termination area earlier than the fork coming from oriC and is arrested at Tus-ter complexes (7, 10), indicated by the grey bar. **B)** Tandem repeat reversion frequencies measured in the presence and absence of the RecA recombinase in cells with only the native *oriC* (*oriC*$^+$) as well as cells that carry the additional ectopic *oriZ* (*oriC*$^+$ *oriZ*$^+$). Data from 33 individual cultures are shown for each strain, generated across 3 independent experiments. An ANOVA test was performed on each group of data points to determine *p* values, which are indicated above the data points. The strains used were DG010 (*oriC*$^+$), DG076 (*oriC*$^+$*ΔrecA*), DG026 (*oriC*$^+$ *oriZ*$^+$) and DG077 (*oriC*$^+$ *oriZ*$^+$ *ΔrecA*). All had the tandem repeat reporter cassette integrated at *yjhR* near position 4.53 Mbp of the chromosome. **C)** Tandem repeat reversion frequencies measured in the presence and absence of the RecG helicase in cells with the additional ectopic *oriZ* (*oriC*$^+$ *oriZ*$^+$). Data from 22 individual cultures are shown for each strain, generated across 2 independent experiments. The strains used were DG026 (*oriC*$^+$ *oriZ*$^+$) and DG069 (*oriC*$^+$ *oriZ*$^+$ *ΔrecG*). All had the tandem repeat reporter cassette integrated at *yjhR* near position 4.53 Mbp of the chromosome.

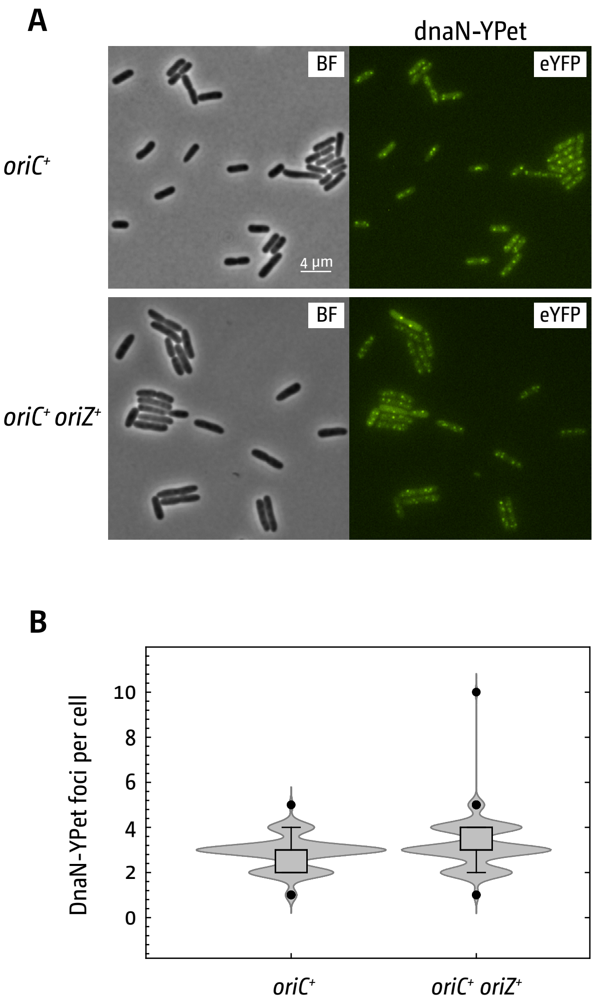

Figure S3: Replication dynamics in exponentially growing cells with one and two origins of replication.
A) Cells were grown to mid exponential growth phase in LB broth (Miller), transferred to an agarose pad (see Material & Methods) and visualised. Active DNA synthesis is highlighted using the replisome sliding clamp protein (DnaN) fused to the fluorophore YPet. The strains used were AS1062 (*oriC*$^{+}$) and RCe749 (*oriC*$^{+}$ *oriZ*$^{+}$). **B)** Foci numbers form the experiment shown in A) were quantified across two independent biological replicates with at least three random frames selected from each replicate and foci numbers pooled. Number of cells analysed was 210 and 206 for the single origin and double origin strain, respectively.

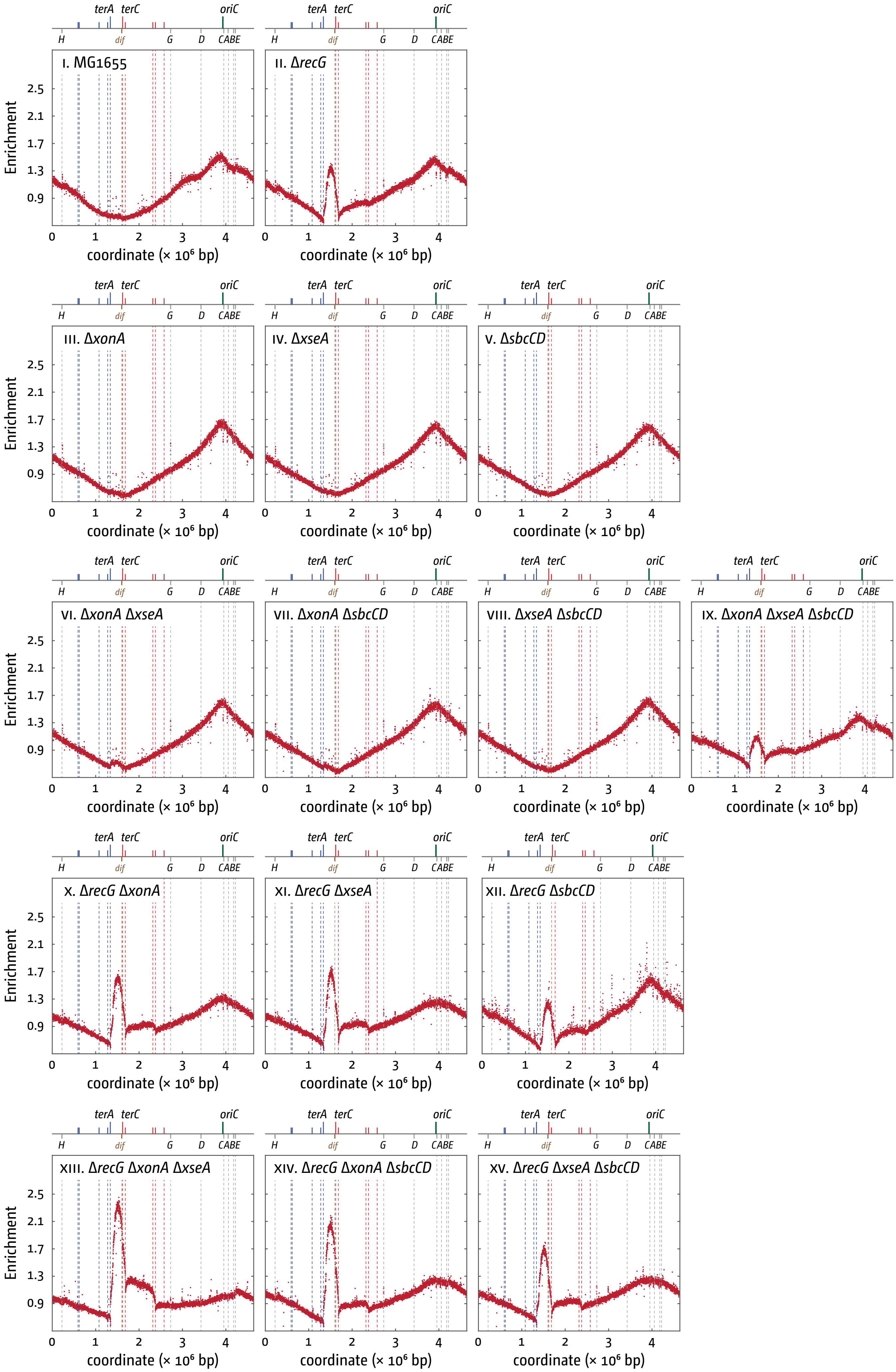

Goodall *et al.* Supplementary Figure 4

Figure S4: Over-replication in the termination area of *E. coli* cells lacking RecG helicase and 3' exonuclease proteins. The number of sequence reads (normalised against reads for a stationary phase wild type control) is plotted against the chromosomal location. A schematic representation of the *E. coli* chromosome showing positions of *oriC* and *ter* sites (above) as well as *dif* and *rrn* operons *A–E*, *G* and *H* (below) is shown above the plotted data. Sequencing templates were isolated from MG1655 (wild type), AU1015 (*ΔrecG*), RCe563 (*ΔxonA*), SLM1185 (*ΔxseA*), RCe562 (*ΔsbcCD*), SLM1203 (*ΔxonA ΔxseA*), JD1017 (*ΔxonA ΔsbcCD*), SLM1209 (*ΔxseA ΔsbcCD*), JD1504 (*ΔxonA ΔxseA ΔsbcCD*), RCe667 (*ΔxonA ΔrecG*), JD1587 (*ΔxseA ΔrecG*), RCe666 (*ΔsbcCD ΔrecG*), JD1579 (*ΔxonA ΔxseA ΔrecG*), JD1577 (*ΔxonA ΔsbcCD ΔrecG*) and JD1576 (*ΔxseA ΔsbcCD ΔrecG*).

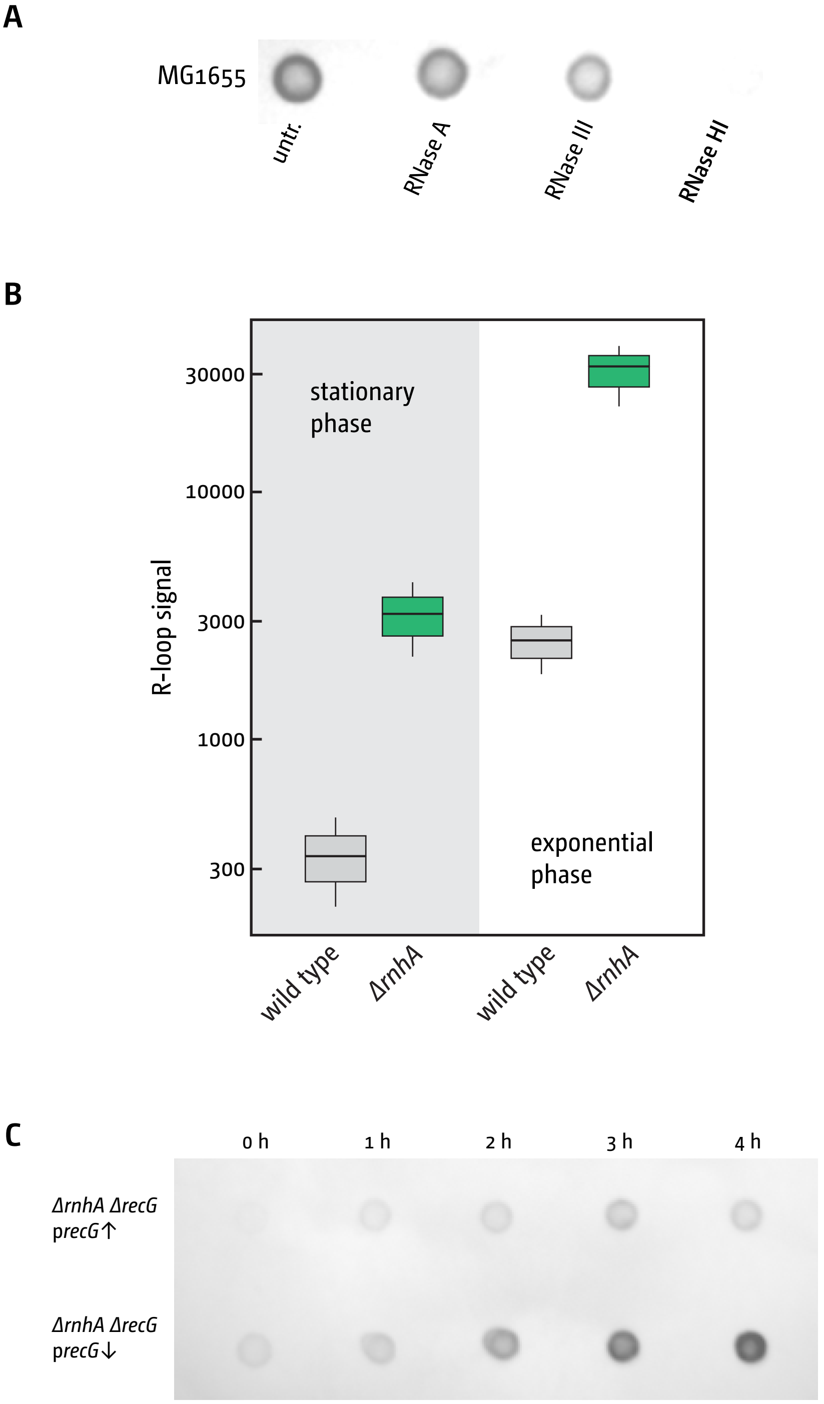

Figure S5: R-loop levels in the presence and absence of RNase HI and RecG helicase and susceptibility of detected structures to various RNases.
A) Genomic DNA was extracted from wild type cells (MG1655) and adjusted to 100 ng/µl to allow detection of the relatively low signal levels. Equal volumes of gDNA were then pre-treated with the RNases indicated for 60 min before the dot blot was performed as described in Material & Methods. The fact that RNase HI removes all of the signal confirms that a significant proportion of the structures recognised by the S9.6-specific antibody are bona fide R-loops. **B)** R-loop levels in exponentially growing and stationary cells. For the exponentially growing samples cells were grown to an A_{600} of 0.4. For the stationary phase samples cells were grown overnight under agitation. Extraction of genomic DNA, dot blotting and R-loop detection using the S9.6 antibody was performed as described in Material & Methods. Shown are the averages of three independent experiments. The strains used were MG1655 (wild type) and AM1974 (*ΔrnhA*). **C)** Detection of R-loops in genomic DNA of cells lacking both RecG helicase and RNase HI. Cells carrying deletions of the genes for RecG helicase and RNase HI, with the deletion of *recG* covered by a plasmid with an arabinose-inducible *recG* gene, were grown in rich medium with arabinose to maintain *recG* expression to early exponential phase. Cells were then washed and resuspended in rich medium with either arabinose (top row) or glucose (bottom row), either maintaining expression of *recG* or switching it off. Samples were taken at the times indicated. Genomic DNA was extracted and the dot blot performed as described in Material & Methods. The strain used was JD1450 (*ΔrnhA ΔrecG* p*recG*).

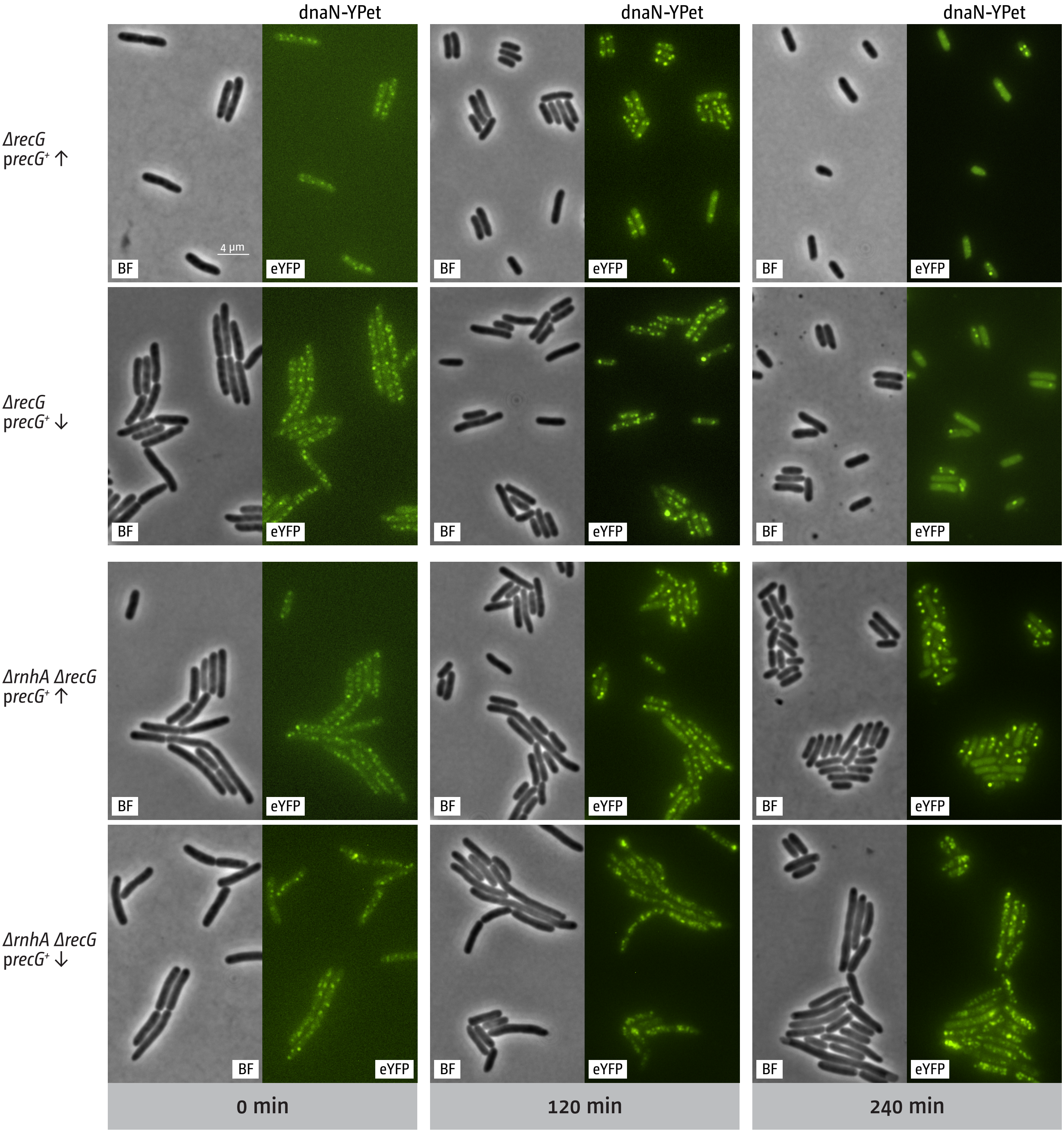

Figure S6. DNA synthesis in cells lacking RecG helicase and RNase HI.
ΔrecG precG and *ΔrecG ΔrnhA precG* cells were grown in rich medium with arabinose to maintain *recG* expression to early exponential phase. Cells were then washed and resuspended in rich medium with either glucose or arabinose, switching the expression of the recG gene either on (upwards pointing arrow) or off (downwards pointing arrow). DNA replication was visualised via a fluorescent fusion of the β sliding clamp with the YFP derivative YPet. Samples were taken at the times indicated. The strains used were RCe941 (*ΔrecG ypet-dnaN precG*) and RCe951 (*ΔrecG ΔrnhA ypet-dnaN precG*). One representative set of images from three independent experimental replicates is shown.

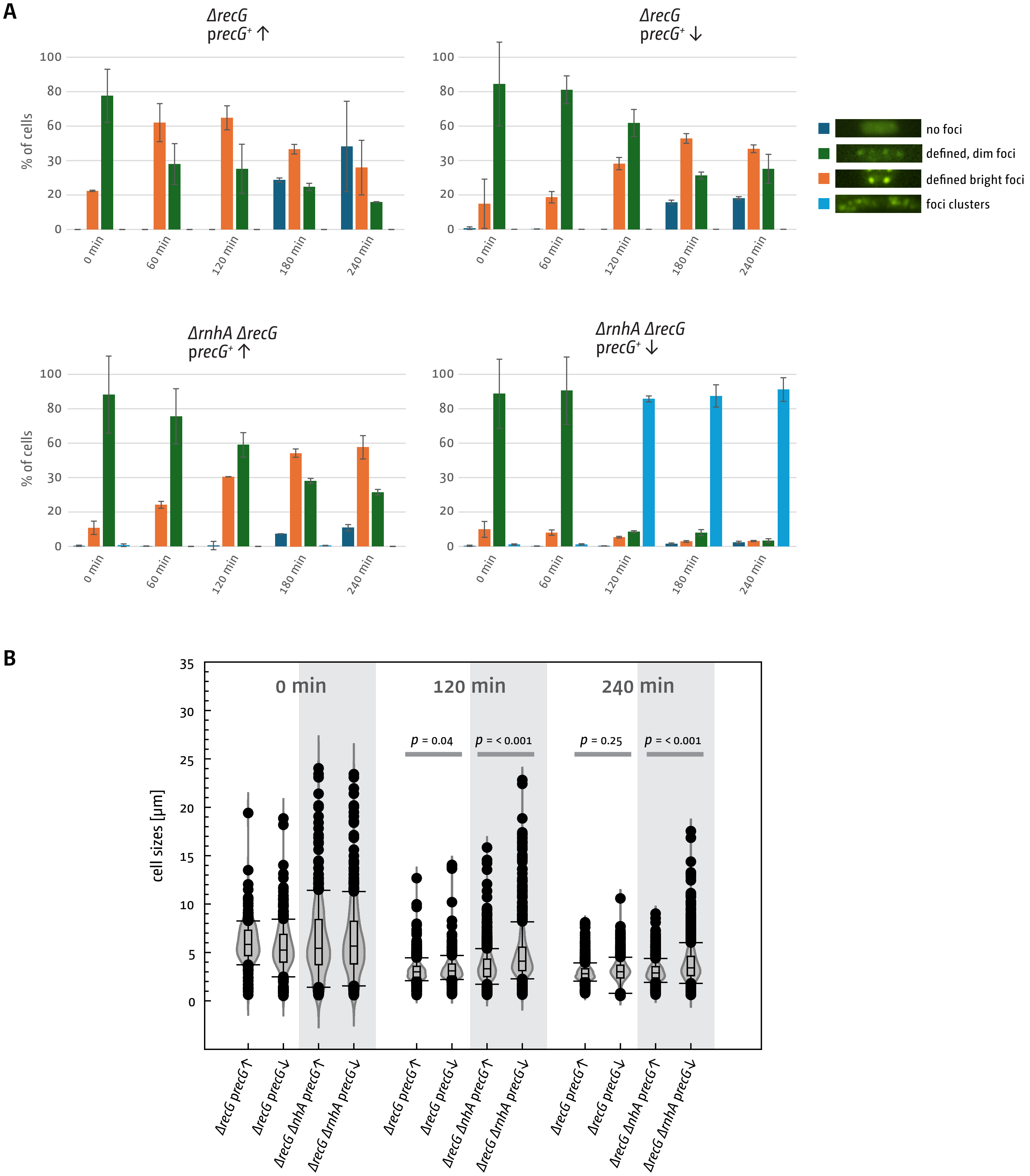

Figure S7. Replisome numbers and cell length analysis in cells lacking RecG helicase and RNase HI.
A) Cells were treated and visualised as described for Figure S6. Across the experiments 4 major types of foci distributions were found as shown in the legend: cells with no foci (dark blue), cells with defined but dim foci (green), cells with defined and bright foci (orange) and cells that contained signal that cannot be resolved as single foci and that therefore was defined as "foci cluster". Cells from a minimum of 3 frames per experiment and time point were analysed. Between 298 and 638 cells were analysed, depending on how many cells were visible across the frames analysed. The strains were as described in Figure S6.
B) Brightfield images as shown in Figure S6 were used for a cell length analysis via Fiji/MicrobeJ (see Material & Methods). As cell density was variable on the images obtained, the number of cells analysed varied between time points and conditions; for the analysis shown we analysed a minimum of 232 and a maximum of 746 cells. Stated *p* values were established via a two-tailed students t-test. The strains were as described in Figure S6.

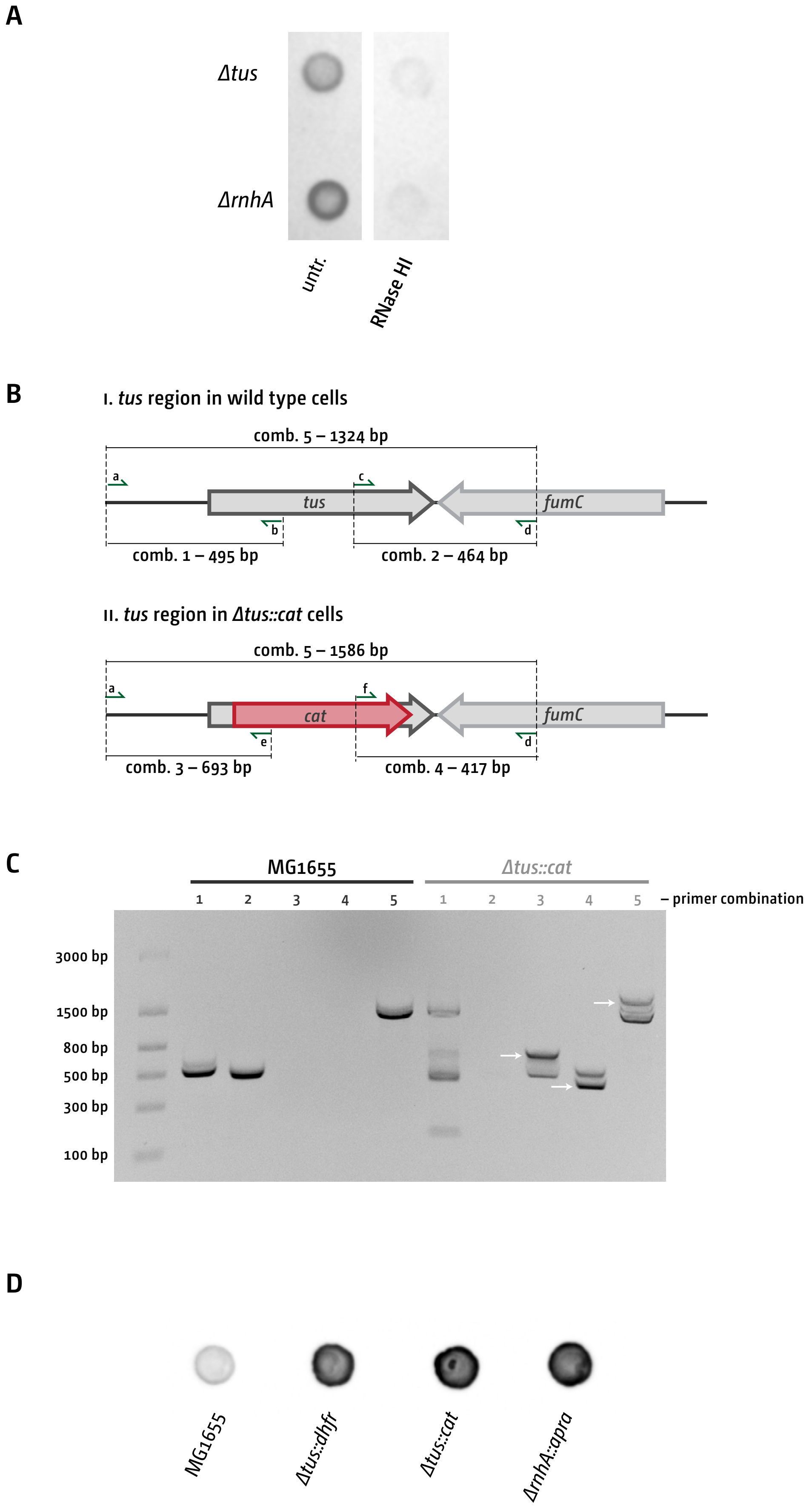

Goodall *et al.* Supplementary Figure 8

Fig. S8: R-loop levels and replication profiles in the presence or absence of Tus terminator protein.
A) Genomic DNA was extracted from cells lacking RNase HI and Tus, respectively. Equal volumes of concentration-adjusted gDNA were then pre-treated with RNase HI for 60 min. The dot blot was performed subsequently as described in Material & Methods. The fact that RNase HI removes all of the signal from both samples confirms that a significant proportion of the structures recognised by the S9.6-specific antibody in these mutants are bona fide R-loops. The strains used were AM1775 (*Δtus*) and AM1974 (*ΔrnhA*). **B)** Schematic representation of the chromosomal region containing the *tus* gene before and after disruption. Primers used for verification of the deletion are shown in green (a – f). The sizes of the PCR products are indicated between the appropriate primer combinations. **C)** PCR verification of the *Δtus::cat* deletion. All strains were tested using 4 primer combinations, thereby testing for the presence or absence of the wild type region as well as the replacement cassette, as indicated in B). In wild type cells only primer combinations a/b and c/d generate a PCR product, which is clearly visible in lanes 1 & 2, while there are no bands in lanes 3 & 4. In the deletion mutants only primer combinations a/e and d/f should generate a specific PCR product, and combination 5 (a combined with d) should show an increased size. PCRs with gDNA from the *Δtus::cat* strain shows some unspecific bands, but the specific wild type bands are clearly absent, and products with the expected sizes are present, as highlighted by the white arrows. This verifies that the wild type copy of the *tus* gene is replaced with the chloramphenicol resistance cassette. **D)** Genomic DNA was extracted from wild type cells, cells lacking RNase HI and two strains with different *tus* alleles, *Δtus::cat* and *tus1::dhfr*. The dot blot was performed as described in Material & Methods. The strains used were MG1655 (wild type), RCe960 (*Δtus::cat*), RCe942 (*tus1::dhfr*) and AM1974 (*ΔrnhA::cat*).

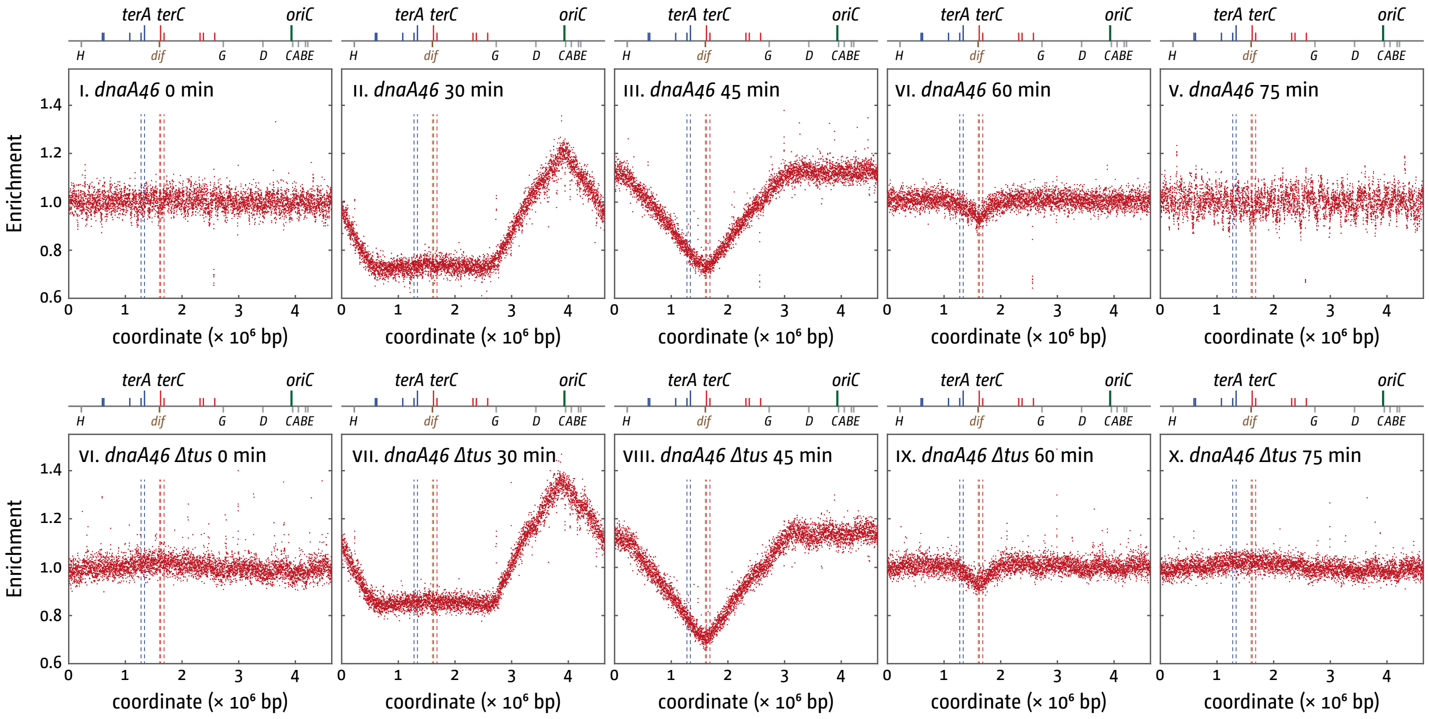

Fig. S9: DNA replication dynamics in synchronised cells in the presence and absence of Tus terminator protein. Cells were grown at 30 °C to early exponential cells. The temperature was then shifted to 42°C for 90 min to block firing of *oriC* while all ongoing rounds of synthesis can be completed. Cultures were then shifted to 30 °C for 30 min to allow the initiation of synthesis at *oriC* before shifting back to 42 °C to prevent further rounds of DNA synthesis. The initial shift to permissive temperature is given as the "0 min" time point. The number of sequence reads (normalised against reads for a stationary phase wild type control) is plotted against the chromosomal location. A schematic representation of the *E. coli* chromosome showing positions of *oriC* and *ter* sites (above) as well as *dif* and *rrn* operons *A–E*, *G* and *H* (below) is shown above the plotted data. Sequencing templates were isolated from AU1054 (*dnaA46*) and RCe203 (*dnaA46 Δtus*).
